# A preliminary study on the insect fauna of Al-Baha Province, Saudi Arabia, with descriptions of two new species

**DOI:** 10.3897/zookeys.274.4529

**Published:** 2013-03-01

**Authors:** Magdi S. El-Hawagry, Mohammed W. Khalil, Mostafa R. Sharaf, Hassan H. Fadl, Abdulrahman S. Aldawood

**Affiliations:** 1 Basic Sciences Department, Community College, Al-Baha University, Al-Baha, Saudi Arabia, PO Box 1598, Project: Survey and Classification of Agricultural and Medical Insects in Al-Baha Province; 2 Plant Protection Department, College of Food and Agriculture Sciences, King Saud University, Riyadh 11451, PO Box 2460, Kingdom of Saudi Arabia

**Keywords:** Palaearctic, Afrotropical, Eremic, List, Insect species, Arabian Peninsula, Tihama, Al-Sarah, Al-Sarawat Mountains, new species

## Abstract

A preliminary study was carried out on the insect fauna of Al-Baha Province, south-western part of Saudi Arabia. A total number of 582 species and subspecies (few identified only to the genus level) belonging to 129 families and representing 17 orders were recorded. Two of these species are described as new, namely: *Monomorium sarawatensis* Sharaf & Aldawood, **sp. n.** [Formicidae, Hymenoptera] and *Anthrax alruqibi* El-Hawagry **sp. n.** [Bombyliidae, Diptera]. Another eight species are recorded for the first time in Saudi Arabia, namely: *Xiphoceriana arabica* (Uvarov, 1922) [Pamphagidae, Orthoptera], *Pyrgomorpha conica* (Olivier, 1791) [Pyrgomorphidae, Orthoptera], *Catopsilia florella* (Fabricius, 1775) [Pieridae, Lepidoptera], *Anthrax chionanthrax* (Bezzi, 1926) [Bombyliidae, Diptera], *Spogostylum* near *tripunctatum* Pallas *in* Wiedemann, 1818 [Bombyliidae, Diptera], *Cononedys dichromatopa* (Bezzi, 1925) [Bombyliidae, Diptera], *Mydas* sp. [Mydidae, Diptera], and *Hippobosca equina* Linnaeus, 1758 [Hippoboscidae, Diptera]. Al-Baha Province is divided by huge and steep Rocky Mountains into two main sectors, a lowland coastal plain at the west, known as “Tihama”, and a mountainous area with an elevation of 1500 to 2450 m above sea level at the east, known as “Al-Sarat or Al-Sarah” which form a part of Al-Sarawat Mountains range. Insect species richness in the two sectors (Tihama and Al-Sarah) was compared, and the results showed that each of the two sectors of Al-Baha Province has a unique insect community. The study generally concluded that the insect faunal composition in Al-Baha Province has an Afrotropical flavor, with the Afrotropical elements predominant, and a closer affiliation to the Afrotropical region than to the Palearctic region or the Eremic zone. Consequently, we tend to agree with those biogeographers who consider that parts of the Arabian Peninsula, including Al-Baha Province, should be included in the Afrotropical region rather than in the Palaearctic region or the Eremic zone.

## Introduction

**Figure 1.**
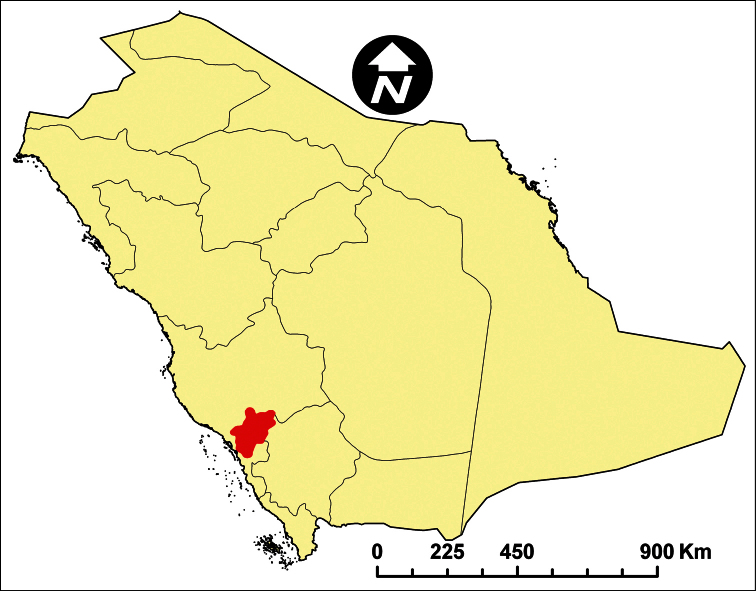
Map of Saudi Arabia showing Al-Baha Province.

Al-Baha Province (Fig. 1) is situated in the south-western part of Saudi Arabia between the Holy Makkah and Asir Regions (Doha 2009), with a population of about 500,000. It is the smallest province in the kingdom of Saudi Arabia (about 10362 km²), situated between longitudes 41°/42° E and latitudes 19°/20° N. This Province is known for its beauty and has many tourist attractions such as forests (about 53 forests), wild life areas, valleys, and mountains. It is characterized by natural tree cover and agricultural plateaus. The region is divided by huge and steep rocky mountains into two main sectors, a lowland coastal plain at the west, known as “Tihama”, and a mountainous area with an elevation of 1500 to 2450 m above sea level at the east, known as “Al-Sarat or Al-Sarah” which form a part of Al-Sarawat Mountains range (Alahmed et al. 2010, and Ibrahim and Abdoon 2005).

Al-Baha Province consists of six main districts, four of which are located in Al-Sarah sector beside the downtown “Al-Baha”, i.e., Al-Aqiq, Al-Mandaq, Al-Qura, and Baljurashi, while two of the districts are located in Tihama sector, namely Al-Mekhwa including Dhee Ain Village (The Marble Village), and Qelwa (website: http://www.albahakfhaa.org/Albaha.htm).

The climate in Al Baha Province is greatly influenced by its varying topography. It is generally moderate in summer and cold in winter with average temperatures ranging between 12–23 °C. In Tihama, the climate is hot in summer, warm in spring and mild in winter, with humidity ranging between 52% - 67%, and a rainfall less than 100 mm annually. While in the mountainous area, Al-Sarah, The climate is greatly different from that in Tihama although the two sectors are separated by no more than 30 km. The weather is cooler in summer and winter due to its high altitude. Al-Sarah is exposed to the formation of clouds and fog, and this often happens in winter because of air masses coming from the Red Sea, accompanied by thunderstorms. In spring and summer, the climate is mild and pleasant. Also, rainfall is higher with falls in the range of 229–581 mm. The average rainfall throughout the whole province is 100–250 mm annually (websites: http://www.tititudorancea.com/z/weather_al_baha_saudi_arabia.htm).

The purpose of this paper is to present a preliminary list of insect fauna in Al-Baha Province. Such a study is of particular interest as the study area is a part of the Arabian Peninsula which is thought by many authors to touch three of the world’s main zoogeographical regions: the Afrotropical, the Palaearctic, and the Oriental (Hölzel 1998).

Many authors agree that the Afrotropical region covers all of Africa south of the Sahara with the island of Madagascar and the nearby smaller islands constituting a distinct subregion. Many authors also include parts of the Arabian peninsula in the Afrotropical region, but there seems to be no agreement as to how much. Sclater (1858) and Wallace (1876) proposed the classical zoogeographical regions and placed the northern border of the Afrotropics along the Tropic of Cancer. Thus, Al-Baha Province was included in the Afrotropical region, and the Northern limit of the Afrotropical region was placed in the Taif area, about 200 km north to Al-Baha (Hölzel 1998). However, according to Uvarov (1938), Greathead (1980), and Larsen (1984) this area should be united with the central Arabian deserts which are either considered part of the Palaearctic, or by some authors as an autonomous Eremic zone (also called the Saharo-Sindian faunal region). All these facts seem to be reflected somehow on the insect faunal composition in Al-Baha Province as shown in the following results.

Undoubtedly, this study is of particular interest also as the insect fauna of Al-Baha Province has not been studied thoroughly before, and this is the first comprehensive study on the entire insect fauna in the region. For this reason, the following previously established data are intended to serve as a basis for further investigations.

Only a few scattered studies have been carried out on select insect groups particularly in Al-Baha (Doha 2009) or have focused on the description of individual species (Aldawood et al. 2011; Lehrer and Abou-Zied 2008; Sharaf and Aldawood 2011, 2012; Sharaf et al. 2012a, 2012b). However, many studies in select insect groups have been carried out in Saudi Arabia as a whole. Many of these studies have been consulted in order to classify the species collected in the current survey or to determine species previously recorded from Al-Baha, and such studies include the following: Abdullah and Merdan (1995), Alahmed et al. (2010), Aldryhim and Khalil (1996), Amoudi (1993), Amoudi and Leclercq (1992), Balkenohl (1994), Basilewsky (1979), Bílý (1979, 1980, 1982, 1985, 1990), Bolton (1976, 1977, 1980, 1995), Boorman (1989), Brown (2000), Bryant (1957), Büttiker (1980), Chassain (1979, 1983), Coiffait (1979), Collingwood (1985), Collingwood and Agosti (1996), Collingwood and van Harten (2005), Collingwood et al. (1997), Collingwood et al. (2004), Cranston and Judd (1989), Crosskey and Buttiker (1982), Daccordi (1979), Damoisseau (1979), Dawah and Abdullah (2006), Decelle (1979), Deeming (1998), Dlabola (1979, 1980), Doguet (1979, 1984), Doha (2009), Español (1981), Fürsch (1979), Fürsch (1979), Gorochov (1993), Greathead (1980, 1988), Guichard (1985, 1986, 1988), Hamid and Hamid (1985), Hölzel (1980, 1982, 1983a, 1983b, 1987, 1988, 1998), Holzschuh (1979), Holzschuh and Téocchi (1991) Horstmann (1981), Ibrahim and Abdoon (2005), Kaltenbach (1982), Kaszab (1979, 1981, 1982), Kwieton (1981), Larsen (1979, 1983, 1984), Leclercq (1982, 1986, 2000), Lewis and Buttiker (1980), Linnavuori (1986), Linnavuori and Alâmy (1982), Lopatin (1979, 1982, 1983), Medvedev (1996), Merz and Dawah (2005), Nagel (1982), Paulian (1980), Pittaway (1985), Pont (1991), Popov (1981a, 1981b), Povolný (1980, 1981, 1983, 1986), Richards (1984), Schawaller (1993), Schawaller et al. (2011), Uhmann (1998), Waterston (1980), Wiltshire (1980, 1982, 1983, 1984, 1986, 1988), Winkler (1981), Würmli (1979), and Zunino (1981).

## Material and methods

Insect material for the present study was collected extensively from different localities in Al-Baha Province, from 2008 to 2012 by the authors using sweeping and aerial nets, bait traps, beating sheets, digging, hand picking, light traps, malaise traps, pitfall traps, sticky traps, tray sifting, and yellow pan traps. Data from specimens preserved in the insect collections and literature records are also taken into consideration.

All taxa are arranged herein in alphabetical order. Localities and date of collection are included for the purpose of mapping distribution and activity periods of species in the study region.

### Abbreviations of museums

**BMNH** Natural History Museum, London, United Kingdom.

**CASC** California Academy of Science Collection, San Francisco, California, USA.

**EFC** Efflatoun collection, Entomology Department, Faculty of Science, Cairo University, Egypt.

**KSMA** King Saud Museum of Arthropods, King Saud University, Riyadh, Kingdom of Saudi Arabia.

**MHNG** Muséum ďHistoire Naturelle, Geneva, Switzerland.

**NHMB** Naturhistorisches Museum, Basel, Switzerland.

**WMLC** World Museum Liverpool, Liverpool, United Kingdom.

## Results

### 

A total number of 582 species and subspecies (few identified only to the genus level) belonging to 129 families and representing 17 orders, have been recorded from Al-Baha Province through the present study as follows:

**Class: Insecta**

**Subclass: Pterygota**

**Division: Exopterygota**

**Order: Odonata**

**Suborder: Anisoptera**

**Family: Aeshnidae**

*Anax parthenope* (Sélys, 1839)

Ghabet Raghdan: Decemper.

Dhee Ain: January-February.

**Family: Libellulidae**

*Trithemis arteriosa* (Burmeister, 1839)

Al-Mekhwa: January-May.

Wadi Turabet Zahran: May.

Dhee Ain: May.

* **Collecting method of specimens of the order Odonata:** Aerial nets.

**Order: Orthoptera**

**Suborder: Caelifera**

**Family: Acrididae**

**Subfamily: Acridinae**

**Tribe: Truxalini**

*Truxalis arabica* Uvarov, 1933

Al-Mekhwa: February.

*Truxalis grandis* Klug, 1830

Al-Mekhwa: March.

*Truxalis longicornis* (Krauss, 1902)

Al-Mekhwa: February.

*Truxalis nasuta* (Linnaeus, 1758)

Al-Mekhwa: February.

*Truxalis procera* Klug, 1830

Al-Mekhwa: February.

**Subfamily Cyrtacanthacridinae**

*Schistocerca gregaria* Forsskal, 1775

Common: April-September.

**Subfamily: Eyprepocnemidinae**

**Tribe: Eyprepocnemidini**

*Heteracris popovi* **(Uvarov, 1952)**

Al-Aqiq: September.

Al-Baha: June.

*Heteracris punctata* (Uvarov, 1936)

Al-Baha: June.

**Subfamily: Gomphocerinae**

*Leva arabica* (Uvarov, 1936)

Baljurashi: May.

*Ochrilidia gracilis* (Krauss, 1902)

Al-Mekhwa: March.

*Ochrilidia* sp.

Al-Mekhwa: March.

*Stenohippus mundus* (Walker, 1871)

Dhee Ain: May.

**Subfamily: Oedipodinae**

**Tribe: Acrotylini**

*Acrotylus patruelis*(Herrich-Schäffer, 1838)

Jebel El-Baher: May-July.

Alhawya: April-July.

**Tribe: Epacromiini**

*Aiolopus simulatrix* (Walker, 1870)

Jebel El-Baher: April–August.

Al-Hawya: April-August.

Ghabet Raghdan: April-July.

Ghabet Shahba: May-July.

Wadi Turabet Zahran: May.

*Aiolopus thalassinus* (Fabricius, 1781)

Al-Mekhwa: April.

**Tribe: Sphingonotini**

*Sphingonotus rubescens* (Walker, 1870)

Jebel El-Baher: April–August.

Al-Hawya: April-August.

Ghabet Raghdan: April-July.

Ghabet Shahba: May-July.

*Sphingonotus savignyi* Saussure, 1884

Jebel El-Baher: April-August.

Al-Hawya: April-August.

Ghabet Raghdan: April-July.

Ghabet Shahba: May-July.

**Tribe: Trilophidiini**

*Trilophidia conturbata* (Walker, 1870)

Al-Mekhwa: March-May.

**Tribe: Unassigned**

*Morphacris fasciata* (Thunberg, 1815)

Al-Mekhwa: March-May.

Wadi Turabet Zahran: May.

**Family: Pamphagidae**

**Subfamily: Porthetinae**

*Xiphoceriana arabica* (Uvarov, 1922) [A new record in Saudi Arabia].

Al-Baha: October-May.

**Family: Pyrgomorphidae**

**Subfamily: Pyrgomorphinae**

**Tribe: Poekilocerini**

*Poekilocerus arabicus* (Uvarov, 1922)

Jebel El-Baher: May-June.

Wadi Turabet Zahran: May.

*Poekilocerus bufonius* (Klug, 1832)

Wadi Turabet Zahran: May.

**Tribe: Pyrgomorphini**

*Pyrgomorpha conica* (Olivier, 1791) [A new record in Saudi Arabia]

Ghabet Raghdan: February.

**Family: Tetrigidae**

**Subfamily: Tetriginae**

**Tribe: Tetrigini**

*Paratettix meridionalis* (Rambur, 1838)

Dhee Ain: May.

Suborder: Ensifera

**Family: Gryllidae**

**Subfamily: Gryllinae**

**Tribe: Gryllini**

*Acheta arabica* Gorochov, 1993

Ghabet Raghdan: April.

*Acheta domesticus* Linnaeus, 1758

Al-Mekhwa: February-August.

Wadi Turabet Zahran: March.

*Gryllus bimaculatus* De Geer, 1773

Al-Baha City: September.

Wadi Turabet Zahran: May.

*Gryllus* sp.

Al-Baha (Jebel El-Baher): December.

**Subfamily: Trigonidiinae**

*Trigonidium cicindeloides* Rambur, 1838

Dhee Ain: May.

**Family: Tettigoniidae**

**Subfamily: Conocephalinae**

**Tribe: Conocephalini**

*Conocephalus arabicus* Uvarov, 1933

Dhee Ain: May.

*Conocephalus* sp.

Dhee Ain: May.

**Subfamily: Tettiginiinae**

**Tribe: Platycleidini**

*Platycleis arabica* Popov, 1981

Al-Baha: June.

Wadi Galla: May.

* **Collecting methods of specimens of the order Orthoptera:** Sweeping and aerial nets were the main methods; however, katydids (Tettigoniidae) and crickets (Gryllidae) were collected using light traps as well.

**Order: Dermaptera**

**Family: Forficulidae**

**Subfamily: Forficulinae**

*Forficula auricularia*Linnaeus, 1758

Wadi Galla: May.

**Order: Embioptera**

**Family: Embiidae**

*Arabembia biarmata* Ross, 1981

Wadi Marwan: ?.

* **Collecting method of specimens of the orders Dermaptera and Embioptera:** Pitfall traps.

**Order: Psocoptera**

**Family: Psocidae**

**Subfamily: Amphigerontiinae**

*Blaste arabica* New, 1979

Al-Mandaq: April.

* **Collecting method of specimens of the order Psocoptera:** Hand picking.

**Order: Isoptera**

**Family: Kalotermitidae**

**Subfamily: Bifiditermitinae**

*Epicalotermes aethiopicus* Silvestri, 1918

Jebel Ibrahim: August.

* **Collecting method of specimens of the order Isoptera:** Digging and hand picking.

**Order: Blattodea**

**Family: Blatellidae**

**Subfamily: Blattellinae**

*Blattella germanica* (Linnaeus, 1767)

All localities: Throughout the year.

**Subfamily: Pseudophyllodromiinae**

*Balta biquandi* Grandcolas, 1994

Wadi Marwani: April.

**Family: Polyphagidae**

*Heterogamisca marmorata* Uvarov, 1936

Wadi Galla: May.

*Heterogamisca* sp.

Al-Baha: September.

* **Collecting methods of specimens of the order Blattodea:** Hand picking and Pitfall traps.

**Order: Mantodea**

**Family: Empusidae**

**Subfamily: Blepharodinae**

*Blepharopsis mendica nuda* Giglio-Tos, 1917

Al-Baha: April.

**Subfamily: Empusinae**

**Tribe: Empusini**

*Empusa spinosa* Krauss, 1902

Al-Baha: June.

**Family: Eremiaphilidae**

*Eremiaphila arabica* Saussure, 1871

Al-Baha: April.

Al-Mekhwa: May-August.

*Eremiaphila* sp

Ghabet Raghdan: May-July.

Ghabet Shahba: May-August.

Jebel El-baher: April-June.

**Family: Mantidae**

**Subfamily: Amelinae**

**Tribe: Amelini**

*Elaea* sp.

Al-Baha City: April.

**Subfamily: Mantinae**

*Hierodula trimacula* Saussure, 1870

Adanan: September.

*Iris coeca* Uvarov, 1931

Adama: September.

*Mimomantis* sp.

Jebel El-Baher: November.

**Subfamily: Miomantinae**

*Eremoplana infelix* Uvarov, 1924

Adanan: September.

*Microthespis dmitriewi* Werner, 1908

Adanan: June-September.

*Rivetina pallida* Kaltenbach, 1984

Al-Baha: April.

**Subfamily: Oxyothespinae**

*Sinaiella nebulosa* Uvarov, 1924

Al-Baha: April.

* **Collecting methods of specimens of the order Mantodea:** Hand picking and Sweeping nets.

**Order: Phthiraptera**

**Suborder: Anoplura**

**Family: Pediculidae**

*Pediculus humanus capitis* De Geer, 1767

All localities: Throughout the year.

**Family: Polyplacidae**

*Polyplax brachyrrhyncha* Cummings, 1915

Adama: September.

* **Collecting method of specimens of the order Phthiroptera:** Hand picking.

**Order: Hemiptera**

**Family: Alydidae**

*Mirperus jaculus* (Thunberg, 1783)

Dhee Ain: May.

Al-Mekhwa: February.

Wadi Turabet Zahran: May.

**Family: Anthocoridae**

**Subfamily: Anthocorinae**

**Tribe: Oriini**

*Orius laevigatus* (Fieber, 1860)

Haraja: February.

**Family: Cydnidae**

*Sehirus tibialis* (Stal, 1853)

Dhee Ain: May.

Wadi Galla: May.

**Family: Dinidoridae**

*Coridius viduatus* (Fabricius, 1794)

Wadi Dahyan: May.

**Family: Lygaeidae**

**Subfamily: Lygaeinae**

*Lygaeus buettikeri* Hamid & Hamid, 1985

Baljurashi: August.

*Spilostethus pandurus (*Scapula, 1763)

Ghabet Raghdan: May-July.

Ghabet Shahba: May-June.

Dhee Ain: May.

Wadi Turabet Zahran: May.

**Subfamily: Orsillinae**

**Tribe: Nysiini**

*Nysius cymoides* (Spinola, 1837)

Al-Baha: February-July.

Wadi Turabet zahran: May.

Dhee Ain: May.

**Subfamily: Oxycareninae**

*Oxycarenus hyalinipennis* (Costa, 1847)

Al-Baha: May-July.

Ghabet Raghdan: May-August.

*Oxycarenus zavattarii* Mancini, 1939

Ghabet Raghdan: May-August.

**Family: Miridae**

**Subfamily: Deraeocorinae**

**Tribe: Deraeocorini**

*Deraeocoris martini* (Puton, 1887)

Zahran: February.

**Subfamily: Mirinae**

**Tribe: Mirini**

*Creontiades pallidus* (Rambur, 1842)

Ghabet Raghdan: May.

*Megacoelum oculare* Wagner, 1957

Adnan: September.

*Phytocoris kansisrob* Linnavouri, 1975

Adama: September.

Zahran: September.

*Taylorilygus pallidulus* (Blanchard, 1852)

Zahran: February.

*Taylorilygus simonyi* (Reuter, 1903)

Al-Mandaq: April.

**Subfamily: Phylinae**

**Tribe: Hallodapini**

*Hallodapus costai* (Reuter, 1890)

Wadi Ganaah: February.

*Laemocoris trimaculatus* Linnavouri, 1964

Al-Mandaq: April.

*Ruwaba glabriceps* Linnavouri &Al-Neamy, 1982

Adama: September.

**Tribe: Phylini**

*Campylomma acaciae* Linnavuori, 1961

Zahran: February.

*Campylomma pulicariae* (Linnavuori, 1986)

Zahran: February.

*Campylomma torridum* Linnavuori, 1975

Zahran: February.

*Psallomimus ornatus* Linnavouri, 1957

Zahran: September.

**Family: Notonectidae**

**Subfamily: Anisopinae**

*Anisops debilis* Gerstäcker, 1873

Wadi Turabet Zahran: May.

*Anisops sardea* Herrich-Schaeffer, 1849

Wadi Turabet Zahran: May.

**Family: Pentatomidae**

**Subfamily: Pentatominae**

**Tribe: Aelini**

*Stenozygum coloratum* (Klug, 1845)

Ghabet Raghdan: May-August.

Wadi Turabet Zahran: April-July.

Dhee Ain: April-September.

**Tribe: Agonoscelidini**

*Agonoscelis arabica* Linnavouri, 1975

Ghabet Raghdan: May.

Wadi Turabet Zahran: June.

**Tribe: Eysarcorini**

*Eysarcoris ventralis* (Westwood, 1837)

Dhee Ain: May.

Wadi Genouna: May.

**Tribe: Pentatomini**

*Acrosternum millieri* (Mulsant & Rey, 1866)

Dhee Ain: May-July.

Wadi Gala: May-June.

Wadi Genouna: May-July.

Wadi Turabet zahran: May-June.

**Tribe: Sciocorini**

*Sciocoris* sp.

Wadi Dahyan: April-July.

Wadi Galla: May-August.

Dhee Ain: May-June.

**Family: Reduviidae**

**Subfamily: Harpactorinae**

*Nagusta simonis* Puton, 1890

Adama: April.

*Sphedanolestes* sp.

Al-Mekhwa: February.

**Subfamily: Peiratinae**

*Pirates strepitans* Rambur, 1839

Wadi Genouna: May.

**Subfamily: Reduviinae**

*Holotrichius innesi* Horvath, 1910

Baljurashi: August.

*Reduvius nanus* Miller, 1951

Wadi Ganaah: February.

**Subfamily: Stenopodainae**

*Pakesia linnavuorii* (Dispons, 1962)

Baljurashi: August.

**Family: Rhopalidae**

**Subfamily: Rhopalinae**

*Liorhyssus hyalinus* (Fabricius, 1794)

Wadi Turabet Zahran: February-May.

**Family: Rhyparochromidae**

**Subfamily: Rhyparochrominae**

**Tribe: Rhyparochromini**

*Dieuches mucronatus*, (Stal, 1866)

Wadi Turabet Zahran: June.

**Family: Scutelleridae**

*Deroplax silphoides* (Thunberg, 1783)

Wadi Dahyan: May.

*Odontoscelis* sp.

Ghabet Shahba: February-March.

* **Collecting methods of specimens of the order Hemiptera:** Beating sheets and sweeping nets were the main methods; however, some specimens of Lygaeidae and Pentatomidae were collected using light traps as well.

**Order: Homoptera**

**Suborder: Auchenorrhyncha**

**Family: Cicadellidae**

**Subfamily: Deltocephalinae**

**Tribe: Athysanini**

*Adama buettikeri* Dlabola, 1980

Al-Baha: September.

*Athysanus* sp.

Al-Mekhwa: February-March.

*Exitianus fasciolatus* (Melichar, 1911)

Wadi Morah: April.

*Paraphlepsius* sp.

Wadi Dhayan: May.

*Texananus* sp.

Wadi Turabet Zahran: May-July.

**Subfamily: Lassinae**

**Tribe: Iassini**

*Batracomorphus* sp.

Dhee Ain: May.

**Subfamily: Ledrinae**

*Petalocephala turgida* Linnavouri, 1962

Wadi Morah: April.

**Subfamily: Macropsinae**

*Macropsis octonotata* Dlabola, 1979

Wadi Turabet Zahran: June.

**Family: Cicadidae**

**Subfamily: Cicadinae**

**Tribe: Cicadini**

*Cicada* sp.

Al-Baha (place and date unknown).

**Family: Cixiidae**

*Pseudoliarus palestinensis* Linnavuori, 1962

Baljurashi (Wadi Marah).

**Family: Dictyopharidae**

**Subfamily: Dictyopharinae**

*Dictyophara* sp.

Wadi Turabet Zahran: June.

**Family: Flatidae**

*Derisa atratula* Melichar, 1902

Dhee Ain: February.

**Family: Nogodinidae**

*Philbyella banajai* Dlabola, 1980

Adama: September.

* Collecting methods of specimens of the order Homoptera: Beating sheets and sweeping nets were the main methods; however, specimens of Cicadellidae, Cicadidae and Cixiidae were collected using light traps as well.

**Division: Endopterygota**

**Order: Neuroptera**

**Family: Chrysopidae**

**Subfamily: Chrysopinae**

**Tribe: Chrysopini**

*Dichochrysa amseli* Holzel,1980

Baljurashi (Wadi Marah): April-May.

*Dichochrysa venosa* (Rambur, 1842)

Baljurashi (Wadi Marah): April-May.

*Mallada spadix* Holzel, 1988

Baljurashi: April.

**Tribe: Belonopterygini**

*Italochrysa asirensis* Hölzel, 1980

Baljurashi (Wadi Marah): April.

**Family: Myrmeleontidae**

**Subfamily: Myrmeleontinae**

**Tribe: Myrmecaelurini**

*Myrmecaelurus acerbus* (Walker, 1853)

Baljurashi: April.

**Tribe: Myrmeleontini**

*Myrmeleon fasciatus* (Navas, 1912)

Baljurashi: April.

*Myrmeleon hyalinus* Olivier, 1811

Al-Baha City: April-July.

**Tribe: Nemoleontini**

*Creoleon antennatus* (Navas, 1914)

Turabet Zahran: April.

*Distoleon laticollis* (Návas, 1913)

Baljurashi: April.

Al-Baha City: April-July.

*Neuroleon lugubris* (Návas, 1926)

Turabet Zahran: April.

**Tribe: Nesoleontini**

*Cueta asirica* (Holzel, 1982)

Baljurashi: April.

*Cueta lineosa* (Rambur, 1842)

Turabet Zahran: April.

*Cueta pallens* (Klug *in* Ehrenberg, 1834)

Turabet Zahran: April.

**Collecting methods of specimens of the order Neuroptera:** Light trap was the main method; however, some specimens of Chrysopidae were collected using sweeping nets as well.

**Order: Coleoptera**

**Suborder: Adephaga**

**Family: Carabidae**

**Subfamily: Brachininae**

**Tribe: Brachinini**

*Brachinus* sp.

Wadi Turabet zahran: June.

*Pheropsophus africanus* (Dejean, 1825)

Wadi Dahyan: May.

**Subfamily: Carabinae**

*Calosoma imbricatum* Klug, 1832

El-Hawya: September.

Wadi Turabet Zahran: May.

**Subfamily: Harpalinae**

**Tribe: Cyclosomini**

*Tetragonoderus arcuatus* Dejean, 1829

Wadi Turabet Zahran: May-June.

Dhee Ain: January-May.

**Tribe: Galeritini**

*Galerita africana* Dejean, 1825

Wadi Ganaah: February.

**Tribe: Harpalini**

*Stenolophus* sp.

Al-Baha: September.

**Tribe: Lebiini**

*Cymindis andreae* Menetries, 1832

Al-Baha: September.

*Cymindis suturalis* Dejean, 1825

Baljurashi (Al-Qamh): January.

**Tribe: Sphodrini**

*Sphodrus leucophthalmus* Linne, 1758

Al-Baha: September.

**Tribe: Zuphiini**

*Agastus zuphoides saudiensis* Mateu, 1986

Wadi Ganaah: February.

**Subfamily: Paussinae**

**Tribe: Paussini**

*Paussus cephalotes* Raffray, 1885

Jebel Shada: April-June.

**Subfamily: Pterostichinae**

**Tribe: Zabrini**

*Amara simplex* Dejean, 1828

Baljurashi (Al-Qamh): January.

*Zabrus* sp.

Ghabet Shahba: February-March.

**Subfamily: Scaritinae**

**Tribe: Clivinini**

*Clivina collaris* (Herbst, 1784)

Dhee Ain: January.

**Subfamily: Trechinae**

**Tribe: Bembidiini**

*Bembidion atlanticum megaspilum* Walker, 1871

Wadi Turabet Zahran: June.

Zee Ghazal: May.

*Elaphropus conspicuous* (Schaum, 1863)

Dhee Ain: January-May.

Wadi El-Zarayeb: May.

*Elaphropus variabilis* (Chaudoir, 1876)

Dhee Ain: January.

*Elaphropus* sp.

Dhee Ain: January.

*Tachys gilvus* Schaum, 1863

Dhee Ain: January.

**Family: Dytiscidae**

**Subfamily: Colymbetinae**

**Tribe: Colymbetini**

*Rhantus includes* (Walker, 1871)

Al-Mandaq: April.

**Subfamily: Dytiscinae**

**Tribe: Dytiscini**

*Hydaticus jucundus* Reiche, 1850

Baljurashi: October.

**Subfamily: Hydroporinae**

**Tribe: Hydroporini**

*Nebrioporus insignis* (Klug, 1834)

Al-Mandaq: April.

*Nebrioporus seriatus* (Sharp, 1882)

Al-Mandaq: April.

**Family: Haliplidae**

*Haliplus lineatocollis* (Marsham, 1802)

Al-Mandaq: April.

Wadi Khoda: November.

**Suborder: Polyphaga**

**Family: Anobiidae**

**Subfamily: Mesocoleopodinae**

*Mesocoelopus ingibbosus* (Pic, 1924)

Adnan: September

**Family: Anthicidae**

**Subfamily: Anthicinae**

**Tribe: Anthicini**

*Anthicus crinitus* LaFerté-Sénectère, 1848

Al-Mekhwa: February-March.

*Stricticollis peplifer* (Marseul, 1879)

Dhee Ain: January.

**Tribe: Endomiini**

*Endomia lefebvrei* (Laferte, 1849)

Al-Aqiq Road: January.

Dhee Ain: January.

**Tribe: Formicomini**

*Anthelephila caeruleipennis* (LaFerté, 1847)

Al-Mekhwa: February-March.

Wadi Turabah: June.

*Anthelephila ninus* LaFerté-Sénectère, 1849

Al-Mekhwa: February-March.

**Family: Bostrichidae**

**Subfamily: Apatinae**

**Tribe: Apatini**

*Xylomedes coronata* (Marseul, 1883)

El-Hawya: September.

**Subfamily: Bostrichinae**

**Tribe: Xyloperthini**

*Enneadesmus trispinosus* (Olivier, 1795)

Wadi Turabet Zahran: June.

*Xyloperthella picea* (Olivier, 1790)

Dhee Ain: August.

**Family: Buprestidae**

**Subfamily: Buprestinae**

**Tribe: Anthaxiini**

*Anthaxia kneuckeri* Obenberger, 1920

Al-Mandaq: September.

**Subfamily: Polycestinae**

*Acmaeodera elevata* (Klug, 1829)

Dhee Ain: May.

*Acmaeodera polita* (Klug, 1829)

El-Hawya: May.

Wadi Galla: May.

**Family: Cerambycidae**

*Mourgliana conspicua* Holzschuh, 1993

Dhee Ain: May.

**Family: Chrysomelidae**

**Subfamily: Bruchinae**

**Tribe: Pachymerini**

*Caryedon* sp.

Al-Mekhwa: February.

**Subfamily: Cryptocephalinae**

**Tribe: Clytrini**

*Aetheomorpha seminigra pumilio* Lacordaire, 1848

Al-Baha: May.

**Tribe: Cryptocephalini**

*Cryptocephalus* sp.

Al-Mekhwa: February.

**Subfamily: Galerucinae**

**Tribe: Alticini**

*Chaetocnema pulla* Chapuis, 1879

Al-Mekhwa: February.

*Chaetocnema tibialis* (Illiger, 1807)

Al-Mekhwa: February.

*Phyllotreta cheiranthi* Weise, 1903

Al-Baha: May.

*Podagrica pallidicolor* Pic, 1909

Wadi Ganaah: February.

*Psylliodes persica* Allard, 1867

Al-Baha: May.

**Tribe: Galerucini**

*Diorhabda octocostata* Gahan, 1896

Ghabet Raghdan: May.

**Family: Cleridae**

**Subfamily: Clerinae**

*Opilo longipilis* Fairmaire, 1892

Wadi Dhyian: September.

**Subfamily: Korynetinae**

*Necrobia rufipes* De Geer, 1775

Wadi Galla: May.

**Family: Coccinellidae**

**Subfamily: Coccinellinae**

**Tribe: Coccinellini**

*Hippodamia variegata* (Goeze, 1777)

Al-Baha: May-June.

Wadi Turabet Zahran: May-June.

**Subfamily: Scymninae**

**Tribe: Scymnini**

*Scymnus syriacus* Marsuel, 1868

Al-Mekhwa: February-April.

**Family: Curculionidae**

**Subfamily: Apioninae**

**Tribe: Apionini**

*Thymapion solarii* (Wagner, 1908)

Jebel Ibrahim: September.

*Thymapion subrecticolle* (Voss, 1961)

Wadi Gaanah: February.

**Tribe: Exapiini**

*Apiotherium dongollanum* (Wagner, 1910)

Jebel Ibrahim: September.

**Tribe: Kalcapiini**

*Afrothymapion tanganum* (Hartmann, 1897)

Jebel Ibrahim: September.

**Tribe: Piezotrachelini**

*Pseudoconapion mirei* (Hoffmann, 1962)

Jebel Ibrahim: September.

*Pseudoconapion segne* (Faust, 1895)

Jebel Ibrahim: September.

**Subfamily: Curculioninae**

**Tribe: Smicronychini**

*Sharpia rubida* (Rosenhauer, 1856)

Al-Baha: May.

**Family: Dryopidae**

*Dryops sulcipennis* (Costa, 1883)

Wadi Turabet Zahran: June.

**Family: Elateridae**

**Subfamily: Agrypninae**

*Lanelater notodonta* (Latreille, 1827)

El-Hawya: September.

**Subfamily: Cardiophorinae**

**Tribe: Cardiophorini**

*Craspedostethus wittmeri* Chassain, 1979

Adnan: August.

**Family: Hydrophilidae**

*Laccobius subpictus erlangeri* (Regimbart, 1905)

Wadi Gaanah: February.

*Laccobius praecipnus* Kuwert, 1891

Al-Mandaq: April.

Khoda: September.

Wadi Gaanah: February.

Wadi Noval: September.

**Family: Meloidae**

**Subfamily: Meloinae**

**Tribe: Mylabrini**

*Mylabris calida* (Pallas, 1782)

Jebel El-Baher: May-July.

Ghabet Shahba: April-August.

Wadi Turabet Zahran: May.

**Subfamily: Nemognathinae**

**Tribe: Nemognathini**

*Nemognatha chrysomelina* (Fabricius, 1775)

Wadi Gala: May.

*Zonitoschema rubricolor* Pic, 1924

Baljurashi: August.

**Family: Melyridae**

*Melyris* sp.

El-Hawya: May.

Dhee Ain: May.

**Family: Mordellidae**

**Subfamily: Mordellinae**

**Tribe: Mordellini**

*Mediimorda bipunctata* (Germar, 1827)

Jebel El-Baher: May-June.

**Family: Mycetophagidae**

**Subfamily: Mycetophaginae**

*Typhaea stercorea* (Linnaeus, 1758)

Dhee Ain: January.

**Family: Prionoceridae**

*Idgia asirensis* Wittmer, 1980

Wadi Gala: May.

Wadi Turabet Zahran: May-October.

**Family: Scarabaeidae**

**Subfamily: Aphodiinae**

**Tribe: Aphodiini**

*Aphodius andreinii* Balthasar, 1939

Wadi Ganaah: February.

*Aphodius lividus* (Olivier, 1789)

Adama: September.

Wadi Ganaah: February.

*Aphodius schusteri* Balthasar, 1935

Wadi Ganaah: February.

**Tribe: Eupariini**

*Ataenius garamas* Peyerimhoff, 1929

Adama: September.

**Tribe: Psammodiini**

*Granulopsammodius plicatulus* (Fairmaire, 1892)

Wadi Al-Uqdah: February.

*Leiopsammodius laevicollis* (Klug, 1845)

Wadi Ganaah: February.

*Rhyssemus Asperocostatus* Fairmaire, 1982

Adanan: September.

Wadi Al-Uqdah: February.

Wadi Ganaah: February.

*Rhyssemus brevitarsis* Pittino, 1984

Wadi Ganaah: February.

*Rhyssemus buettikeri* Pittino, 1984

Wadi Ganaah: February.

*Rhyssemus coluber* Mayet, 1887

Wadi Ganaah: February.

Wadi Shumran: February.

*Rhyssemus granosus* (Klug & Erichson, 1842)

Adama: September.

Adanan: September.

Wadi Ganaah: February.

Wadi Shumran: February.

Dhee Ain: October.

*Rhyssemus rubeolus* Harold, 1871

Wadi Ganaah: February.

*Rhyssemus saoudi* Pittino, 1984

Adama: september.

Adanan: September.

Dhee Ain: May.

**Subfamily: Cetoniinae**

*Homothyrea thoracica* Schaum, 1841

Al-Aqiq Road: January.

Dhee Ain: January.

*Pachnoda leclercqi* Rigout, 1985

Wadi Galla: May.

*Pachnoda thoracica* Fabricius, 1775

Ghabet Shahba: May-December.

Wadi Turabet Zahran: May.

Dhee Ain: May.

**Subfamily: Scarabaeinae**

*Onthophagus transcaspicus* Koenig, 1888

Adnan: August-September.

Baljurashi: August-September.

**Family: Scirtidae**

**Subfamily: Scirtinae**

*Cyphon laevipennis* Tournier, 1868

Wadi Turabet Zahran: July-October.

**Family: Silvanidae**

**Subfamily: Silvaninae**

*Oryzaephilus surinamensis* (Linnaeus, 1758)

Dhee Ain: May.

**Family: Staphylinidae**

**Subfamily: Paederinae**

**Tribe: Paederini**

*Paederus alfierii* Koch, 1934

Dhee Ain: April-June

Wadi Galla: May.

*Paederus* sp.

Al-Mekhwa: February.

**Family: Tenebrionidae**

**Subfamily: Alleculinae**

**Tribe: Alleculini**

*Mycetocharina wittmeri* Muche, 1982

Adnan: September.

*Prionychus denticulatus* Muche, 1982

Adnan: September.

**Subfamily: Pimeliinae**

**Tribe: Adesmiini**

*Adesmia cancellata cancellata* **(**Klug, 1830)

Al-Baha: September.

Adnan: September.

**Tribe: Stenosini**

*Stenosis comata* Reiche & Saulcy, 1857

Baljurashi (Al-Qama’): January

**Tribe: Pimeliini**

*Thriptera crinita* Klug, 1830

Al-Baha City (El-Hawya): September.

Wadi Galla: May.

Wadi Turabet Zahran: May.

*Thriptera kraatzi* Haag, 1876

Dhee Ain: January.

**Tribe: Sepidiini**

*Sepidium cristatum* Fabricius, 1775

Baljurashi: August.

Subfamily: Tenebrioninae

**Tribe: Blaptini**

*Blaps kollari kollari* Seidlitz, 1896

Adnan: September.

**Tribe: Opatrini**

*Anemia brevicollis* (Wollaston, 1864)

Wadi Turabet Zahran: May.

*Gonocephalum strigosum* (Reiche, 1850)

Al-Aqiq Road: January.

**Family: Thanerocleridae**

*Thaneroclerus buqueti* (Lefebvre, 1835)

Ghabet Amadan: May.

**Family: Zopheridae**

**Subfamily: Colydiinae**

**Tribe: Synchitini**

*Bitoma sicciana* (Pascoe, 1863)

Wadi Al-Zarayeb: April.

* **Collecting methods of specimens of the order Coleoptera:** Pitfall traps, especially for Carabidae and Tenebrionidae; beating sheets, especially for Anobiidae and Curculionidae; and sweeping nets, especially for Chrysomelidae, Cerambycidae, Buprestidae and other families were the main methods; however, specimens of Dytiscidae were collected using light traps.

**Order: Trichoptera**

**Family: Hydroptilidae**

**Subfamily: Hydroptilinae**

**Tribe: Hydroptilini**

*Hydroptila cruciata* Ulmer, 1912

Wadi Ilyab: November.

**Family: Leptoceridae**

**Subfamily: Leptocerinae**

**Tribe: Setodini**

*Setodes alalus* Mosely, 1948

Wadi Arida: September.

Wadi Ganaah: February.

Wadi Ilyab: November.

**Family: Philopotamidae**

**Subfamily: Chimarrinae**

*Chimarra saudia* Malicky, 1986

Wadi Arida: September.

* **Collecting methods of specimens of the order Trichoptera:** Light traps.

**Order: Lepidoptera**

**Suborder: Rhopalocera**

**Family: Hesperidae**

**Subfamily: Hesperiinae**

*Pelopidas thrax thrax* (Hubner, 1821)

Al-Mikhwa: January-April.

**Family: Lycaenidae**

**Subfamily: Lycaeninae**

**Tribe: Lycaenini**

*Lycaena phlaeas* (Linnaeus, 1761)

Ghabet Raghdan: April-August.

**Subfamily: Polyommatinae**

**Tribe: Lycaenesthini**

*Anthene* sp.

Al-Baha: June.

**Tribe: Polyommatini**

*Azanus* sp.

Wadi Turabet Zahran: November.

*Euchrysops osiris* (Hopffer, 1855)

Ghabet Shahba: May-June.

*Lepidochrysops pittawayi* Larsen, 1983

Adnan: February-April.

*Tarucus theophrastus* Fabricius, 1793

Al-Mikhwa: January-March.

Wadi Turabet Zahran: November.

*Zizula hylax* Fabricius, 1775

Al-Mikhwa: January-March.

**Subfamily: Theclinae**

*Myrina silenus* (Fabricius, 1775)

Dhee Ain: February-March.

**Family: Nymphalidae**

**Subfamily: Charaxinae**

**Tribe: Charaxini**

*Charaxes bernstorffi* Rydon, 1982

Ghabet Shahba: May.

*Charaxes hansali* Felder, 1867

Ghabet Raghdan: May-June.

**Subfamily: Danainae**

*Danaus chrysippus* (Linnaeus, 1758)

Al-Mekhwa: January-March.

Dhee Ain: June-Novenber.

Dhee Ain: October.

**Subfamily: Heliconiinae**

*Argynnis* sp.

Dhee Ain: December-January.

**Subfamily: Nymphalinae**

**Tribe: Junoniini**

*Junonia hierta* Fabricius, 1798

Ghabet Shahba: May-July.

**Tribe: Nymphalini**

*Vanessa (Cynthia) cardui* Linnaeus, 1758

Al-Baha (Jebel El-Baher): March-July.

Ghabet Raghdan: March-July.

Wadi Turabet Zahran: November.

**Subfamily: Satyrinae**

*Lasiommata felix* (Warnecke, 1929)

Ghabet Shahba: May.

**Family: Papilionidae**

**Subfamily: Papilioninae**

*Papilio demoleus demoleus* Linnaeus, 1758

Al-Mekhwa: March-April.

Dhee Ain: January.

Wadi Turabet Zahran: December.

*Papilio* sp.

Ghabet Raghdan: May-June.

**Family: Pieridae**

**Subfamily: Coliadinae**

*Catopsilia florella* (Fabricius, 1775) [A new record in Saudi Arabia]

Al-Mekhwa: November.

*Eurema hecabe* (Linnaeus, 1758)

Wadi Turabet Zahran: October.

**Subfamily: Pierinae**

**Tribe: Anthocharini**

*Euchloe belemia* (Esper, 1800)

Amadan: October.

**Tribe: Colotini**

*Colotis amata* (Fabricius, 1775)

Al-Mekhwa: January-March.

Dhee Ain: January.

*Colotis antevippe zera* (Lucas, 1852)

Al-Mekhwa: February-Maech.

Dhee Ain: February-March.

*Colotis daira* (Klug, 1829)

Al-Mekhwa: November.

*Colotis danae* (Fabricius, 1775)

Al-Mekhwa: January-February.

Dhee Ain: October.

*Colotis ephyia* (Klug, 1829)

Al-Mekhwa: November.

*Colotis eucharis* Fabricius, 1775

Dhee Ain: March.

*Colotis evagore* (Klug, 1829)

Al-Mekhwa: November.

*Colotis halimede* (Klug, 1829)

Dhee Ain: October.

*Colotis liagore* (Klug, 1829)

Dhee Ain: October-December.

*Colotis protomedia* (Klug, 1829)

Al-Mekhwa: March.

Ghabet Raghdan: May.

*Nepheronia buquetii* (Boisduval, 1836)

Al-Mekhwa: March.

Dhee Ain: February-June.

**Tribe: Pierini**

*Belenois aurota* (Fabricius, 1793)

Al-Baha (Jebel El-Baher): May-June.

Ghabet Raghdan: May-July.

*Madais fausta fausta* (Olivier, 1804)

Dhee Ain: Fabruary.

*Pieris krueperi* (de Niceville, 1884)

Amadan: October.

*Pieris rapae* (Linnaeus, 1758)

Al-Mekhwa: January – June.

Al-Baha: March – August.

Dhee Ain: February – July.

*Pinacopteryx eriphia* (Godart, 1819)

Aqabet Al-Baha-Tihama: April-May.

*Pontia daplidice daplidice* Linnaeus, 1756

Ghabet Raghdan: May-July.

*Pontia glauconome* (Klug, 1829)

Ghabet Raghdan: May-October.

**Suborder: Heterocera**

**Family: Arctiidae**

**Subfamily: Arctiinae**

*Apisa canescens Arabica* Warnecke, 1934

Baljurashi: August.

*Hyphantria cunea* Drury, 1773

Al-Baha (G. El-Baher): May.

*Utetheisa pulchella* (Linnaeus, 1758)

Al-Mekhwa: March.

**Subfamily: Lithosiinae**

*Pelosia arabica* (Rebel, 1907)

Baljurashi: September.

*Siccia arabica* Wiltshire, 1983

Baljurashi: August.

**Family: Carposinidae**

*Metacosmesis xerostola* Diakonoff, 1983

Baljurashi (Wadi Marah): April.

**Family: Choreutidae**

*Tebenna micalis* Mann, 1857

Baljurashi (Wadi Marah): September.

**Family: Cossidae**

*Eremocossus vaulogeri jordana* (Staudinger, 1897)

Baljurashi: September.

*Mormogystia reibellii* (Oberthür, 1876)

Adnan: May.

**Family: Gelechiidae**

**Subfamily: Gelechiinae**

**Tribe: Gelechiini**

*Ephysteris promptella* Staudinger, 1859

Baljurashi (Wadi Marah): April.

*Ephysteris subdiminutella* Stainton, 1867

Baljurashi (Wadi Marah): April.

*Phthorimaea operculella* Zeller, 1873

Baljurashi (Wadi Marah): April.

*Scrobipalpa asiri* Povolny, 1980

Baljurashi (Wadi Marah): April.

*Scrobipalpa biljurshi* Povolny, 1980

Baljurashi (Wadi Marah): April.

*Scrobipalpa ergasima* (Meyrick, 1916)

Baljurashi (Wadi Marah): April.

*Scrobipalpa vicaria* (Meyrick, 1921

Baljurashi (Wadi Marah): April.

**Family: Geometridae**

**Subfamily: Ennominae**

*Cleora pavlitzkiae* Fletcher, 1958

Baljurashi: April.

*Coenina collenettei* Prout, 1931

Baljurashi: September.

*Epigynopteryx guichardi* Wiltshire, 1982

Baljurashi: August.

*Odontopera integraria* Guenée, 1858

Baljurashi: August-September.

*Oreometra fifae* Wiltshire, 1986

Baljurashi: April.

*Xylopteryx guichardi* Wiltshire, 1982

Baljurashi: September.

*Zamarada hyalinaria* Guenée, 1858

Baljurashi: September.

*Zeuctoboarmia syntropha* (Prout, 1931)

Baljurashi: September.

**Subfamily: Geometrinae**

*Microloxia herbaria* Hübner, 1808

Jebel Ibrahim: August-September.

*Prasinocyma eremica* Wiltshire, 1980

Baljurashi: September.

**Subfamily: Larentiinae**

*Calliclystis lita* (Prout, 1916)

Baljurashi: April.

*Chloroclystis hawkinsi* Wiltshire, 1982

Baljurashi: September.

*Orthonama obstipata* (Fabricius, 1794)

Baljurashi: September.

**Subfamily: Sterrhinae**

*Chlorerythra rubriplaga sinaica* Wiltshire, 1949

Baljurashi: September.

*Idaea hesuata* Wiltshire, 1983

Baljurashi: September.

*Idaea sordida sordida* (Rothschild, 1913)

Al-Mandaq: September.

*Rhodometra kikiae* Wiltshire, 1982

Jebel Ibrahim: September.

*Scopula luridata* Zeller, 1847

Baljurashi: April.

Jebel Ibrahim: September.

*Scopula sarfaitensis* Wiltshire, 1982

Baljurashi: April.

*Traminda rufistrigata* Hampson, 1896

Jebel Ibrahim: August-September.

*Traminda neptunaria* Guenée, 1858

Baljurashi: June.

**Family: Lasiocampidae**

*Dendrolimus lendereri* Kocak, 1981

Baljurashi: September.

*Pachypasa sultani* Wiltshire, 1986

Baljurashi: April.

*Stoermeriana omana* Freina&Witt, 1988

Baljurashi: April.

*Streblote acaciae* Klug, 1829

Baljurashi: April.

**Family: Limacodidae**

*Coenobasis farouki* Wiltshire, 1947

Baljurashi: August.

**Family: Lymantriidae**

**Subfamily: Lymantriinae**

**Tribe: Lymantriini**

*Euproctis fasciata* Walker, 1855

Ratha: August.

*Laelia xyleutis* Hampson, 1905

Baljurashi: April-September.

*Lymantriades arabica* (Hampson, 1910)

Baljurashi: August-September.

*Naroma varipes* Walker, 1865

Baljurashi: September.

**Family: Noctuidae**

**Subfamily: Acontiinae**

*Ozarba atrifera* Hampson, 1910

Baljurashi: August.

**Subfamily: Acronictinae**

*Ariathisa abyssinia* Guenée, 1852

Baljurashi: August.

**Subfamily: Bryophilinae**

*Cryphia pittawayi* Wiltshire, 1986

Baljurashi: September.

**Subfamily: Catocalinae**

*Antarchaea magalium* Townsend, 1958

Baljurashi: July.

*Hypotacha ochribasalis* Hampson, 1896

Adnan: September.

*Lyncestis mimica* Gaede, 1939

Baljurashi: August.

*Scodionyx mysticus* Staudinger, 1899

Baljurashi: April.

*Sphingomorpha chlorea* Cramer, 1777

Baljurashi: April.

*Thria robusta* Walker, 1857

Baljurashi: November.

*Ophiuche masurialis* Guenée, 1854

Wadi Gaanah: February.

**Subfamily: Erebinae**

*Tathorhynchus philbyi* Wiltshre, 1986

Baljurashi: July.

**Subfamily: Eriopinae**

*Callopistria latreillei* (Duponchel, 1827)

Baljurashi: July.

Wadi Al-Uqdah: February.

**Subfamily: Eustrotiinae**

*Eublemma bifasciata* (Moore, 1881)

Wadi Al-Uqdah: February.

*Eublemma buettikeri* Wiltshire, 1980

Baljurashi: September.

*Eublemma ecthaemata* Hampson, 1896

Baljurashi: September.

*Eublemma khalifa nejdi* (Wiltshire, 1961)

Baljurashi: September.

*Eublemma mesophaea* Hampson, 1910

Jebel Ibrahim: September.

*Eublemma parva* (Hübner, 1808)

Al-Baha: August.

Jebel Ibrahim: September.

**Subfamily: Hadeninae**

*Agrotis herzogi* Rebel, 1911

Al-Baha: January-June.

*Agrotis ipsilon* (Hufnagel, 1766)

Al-Baha: January-June.

Al-Mekhwa: December-February.

*Agrotis medioatra* Hampson, 1918

Baljurashi: September.

*Caradrina aldegaitheri* Wiltshire, 1986

Baljurashi: September.

*Caradrina localis* Wiltshire, 1986

Baljurashi: September.

Bani Sar: February.

*Caradrina stenoeca*Wiltshire, 1986

Baljurashi: September.

*Haderonia proximoides* Wiltshire, 1982

Baljurashi: September.

*Mythimna affinis* (Warnecke, 1930)

Baljurashi: July.

*Mythimna octogesima* Wiltshire, 1982

Baljurashi: August.

*Sideridis chersotoides* Wiltshire, 1956

Baljurashi: September.

*Spodoptera cilium* Guenée, 1852

Baljurashi: September.

*Spodoptera exigua* (Hubner, 1808)

Al-Baha: January.

*Spodoptera littoralis* (Boisduval, 1833)

Al-Baha: February-July.

Ghabet Raghdan: May-June.

Al-Mekhwa: December-April.

*Spodoptera mauritia* (Boisduval, 1833)

Al-Baha: February-July.

**Subfamily: Plusiinae**

*Trichoplusia vittata* (Wallengren, 1856)

Beljurashi: July.

**Subfamily: Thiacidinae**

*Thiacidas adnanensis* (Wiltshire, 1980)

Adnan: September.

*Thiacidas cerurodes cerurodes* (Hampson, 1916)

Al-Baha: September.

**Family: Oecophoridae**

**Subfamily: Depressariinae**

*Agonopterix subpropinquella* Stainton, 1849

Baljurashi (Wadi Marah): April.

*Depressaria discipunctella* Herrich-Schäffer, 1854

Baljurashi (Wadi Marah): April.

**Subfamily: Unassigned**

*Amseloecia arabica* Povolny, 1983

Baljurashi (Wadi Marah): April.

**Family: Pterophoridae**

**Subfamily:** **Agdistinae**

*Agdistis obstinata* Meyrick, 1920

Baljurashi (Wadi Marah): April.

**Subfamily: Pterophorinae**

**Tribe:** **Oxyptilini**

*Megalorhipida defectalis* Walker, 1864

Baljurashi: May.

*Stangeia siceliota* (Zeller, 1847)

Baljurashi (Wadi Marah): April.

**Family: Scythrididae**

*Catascythris keberella* Amsel, 1935

Baljurashi (Wadi Marah): April.

**Family: Sphingidae**

**Subfamily: Macroglossinae**

**Tribe: Macroglossin**i

*Daphnis nerii* (Linnaeus, 1758)

Al-Baha (Jebel El-Baher): May.

*Hippotion celerio* (Linnaeus, 1758)

Al-Baha: May.

Dhee Ain: April.

*Hyles livornica* (Esper, 1780)

Al-Baha (Jebel El-Baher): May-June.

Ghabet Raghdan: June.

Al-Mandaq: May.

Al-Mekhwa: April-June.

**Subfamily: Sphinginae**

**Tribe: Acherontiini**

*Acherontia atropos* (Linnaeus, 1758)

Al-Baha (El-Hawya): October.

*Agrius convolvuli* (Linnaeus, 1758)

Al-Baha: November-April.

**Tribe: Sphingini**

*Macropoliana asirensis* Wiltshire, 1980

Al-Baha: February.

**Family: Symmocidae**

*Apiletria asirica* Gozmany, 1982

Baljurashi (Wadi Marah): April.

**Family: Thaumetopoeidae**

*Thaumetopoea jordana* Staudinger, 1895

Jebel Ibrahim: September.

**Family: Tineidae**

**Subfamily: Hapsiferinae**

*Hapsifera punctata* Petersen, 1961

Baljurashi (Wadi Marah): April.

*Hapsiferona glareosa* Meyrick, 1912

Baljurashi (Wadi Marah): April.

**Subfamily: Perissomasticinae**

*Neoepiscardia islamella* Petersen & Gaedike, 1982

Baljurashi (Wadi Marah): April.

*Perissomastix amseli* (Petersen, 1959)

Baljurashi (Wadi Marah): April.

*Perissomastix asiriella* Petersen & Gaedike, 1982

Baljurashi (Wadi Marah): April.

*Perissomastix nigriceps* Warren & Rothschild, 1905

Baljurashi (Wadi Marah): April.

**Family: Tortricidae**

**Subfamily: Olethreutinae**

**Tribe: Eucosmini**

*Strepsicrates cryptosema* Diakonoff, 1983

Baljurashi (Wadi Marah): April.

**Tribe: Grapholitini**

*Cydia dissulta* Diakonoff, 1983

Baljurashi (Wadi Marah): April.

*Cydia melanoptycha* Diakonoff, 1983

Baljurashi (Wadi Marah): April.

*Selania resedana* (Obraztsov, 1959)

Baljurashi (Wadi Marah): April.

**Tribe: Olethreutini**

*Eccopsis wahlbergiana* Zeller, 1852

Baljurashi (Wadi Marah): August.

**Subfamily: Tortricinae**

**Tribe: Archipini**

*Procrica ammina* Diakonoff, 1983

Baljurashi (Wadi Marah): August.

*Tebenna micalis* (Mann, 1857)

Baljurashi (Wadi Marah): April.

**Family: Zygaenidae**

**Subfamily: Zygaeninae**

*Reissita simonyi* (Rebel, 1899)

Al-Mikhwa: March-May.

* **Collecting methods of specimens of the order Lepidoptera:** Aerial nets for butterflies (suborder: Rhopalocera), and light traps for moths (suborder: Heterocera).

**Order: Diptera**

**Suborder: Nematocera**

**Family: Ceratopogonidae**

*Culicoides kingi* (Austen, 1912)

Al-Mekhwa: May.

Bejurashi: June.

*Culicoides newsteadi* Austen, 1921

Al-Mekhwa: May.

Beni Hassan: June.

*Culicoides oxystoma* Kieffer, 1910

Ghabet Raghdan: September.

**Family: Chironomidae**

**Subfamily: Tanypodinae**

*Procladius (Holotanypus ) apicalis* (Kieffer, 1918)

Wadi Al-Uqdah: February-March.

Wadi Diyhan: March.

Wadi Shumrukh: April.

*Ablabesmyia (Ablabesmyia) longistyla* Fittkau, 1962

Adnan: September.

Wadi Diyhan: March.

Wadi Ilyab: March.

*Conchapelopia trifascia* (Freeman, 1954)

Adnan: September.

*Larsia rutsburuiemis* (Goetghebuer, 1935)

Al-Mandaq: April.

*Larsia teesdalei* (Freeman, 1955)

Wadi Ilyab: February.

*Paramerina vaillanti* Fittkau, 1962

Wadi Ibrahim: August.

Al-Mandaq: April.

**Subfamily: Orthocladiinae**

*Paraphaenocladius impensus* (Walker, 1856)

Wadi Diyhan: March.

**Subfamily: Chironominae**

**Tribe: Chironomini**

*Dicrotendipes peringueyanus* Kieffer, 1924

Adnan: September.

*Dicrotendipes sudanicus* (Freeman, 1957)

Adnan: September.

Wadi Diyhan: March.

*Paratendipes nubilipennis* Freeman, 1957

Adnan: September.

Wadi Ibrahim: August.

*Paratendipes nudisquama* (Edwards, 1929)

Adnan: September.

Wadi Diyhan: March.

Wadi Ilyab: February.

*Polypedilum (Pentapedilum) wittei* Freeman, 1955

Al-Foqa: September.

*Polypedilum (Polypedilum) buettikeri* Cranston, 1989

Wadi Ilyab: February.

*Polypedilum (Polypedilum) tana* Cranston and Judd, 1989

Adnan: September.

*Stictochironomus puripennis* (Kieffer, 1921)

Jebel Ibrahim: September.

Wadi Ilyab: February.

**Tribe: Tanytarsini**

*Cladotanytarsus pseudomancus* (Goetghebuer, 1934)

Al-Mandaq: April.

Al-Baha: February.

*Cladotanytarsus reductus* (Freeman, 1954)

Adnan: September.

Jebel Ibrahim: September.

*Rheotanytarsus ringei* Lehmann, 1970

Wadi Ilyab: February.

*Tanytarsus mcmillani* Freeman, 1958

Wadi Diyhan: March.

*Tanytarsus trifidus* Freeman, 1958

Wadi Diyhan: March.

Wadi Ibrahim: August.

*Virgatanytarsus nigricornis* (Goetghebuer, 1935)

Wadi Diyhan: March.

**Family: Corethrellidae**

*Corethrella buettikeri* Cranston, 1980

Adnan (W. Iwrakh): September.

**Family: Culicidae**

**Subfamily: Anophelinae**

*Anopheles multicolor* Cambouliu, 1902

All regions of Al-Baha: Throughout the year.

*Anopheles sergentii* (Theobald, 1907)

Al-Baha: June - August.

Al-Mandaq: July.

**Subfamily: Culicinae**

*Aedes caspius* (Pallas, 1771)

Al-Mekhwa: Throughout the year.

Al-Baha: Throughout the year.

*Aedes vittatus* (Bigot, 1861)

Al-Baha: Throughout the year.

*Culex pipiens* Linnaeus, 1758

All regions of Al-Baha: Throughout the year.

**Family: Psychodidae**

**Subfamily: Phlebotomine**

*Phlebotomus (Paraphlebotomus) alexandri* Sinton, 1928

Al-Dafeer: April to August.

Al-Mandaq: March to November.

*Phlebotomus (Adlerius) arabicus* Theodor, 1953

Al-Baha: April to December.

Al-Dafeer: April to December.

Al-Mandaq: June to December.

Al-Mekhwa: July to December.

*Phlebotomus (Phlebotomus) bergeroti* Parrot, 1934

All localities: March to December.

*Phlebotomus (Larroussius) orientalis* Parrot, 1936

Al-Mekhwa: October to December.

*Phlebotomus (Phlebotomus) papatasi* (Scopoli, 1786)

Al-Dafeer: April to December.

*Phlebotomus (Paraphlebotomus) sergenti* Parrot, 1917

Al-Aqiq: April to November.

Al-Baha: April to December.

Al-Dafeer: April to December.

Al-Mekhwa: May to November.

*Sergentomyia (Sergentomyia) antennata* (Newstead, 1912)

Al-Mandaq: April.

*Sergentomyia (Sintonius) clydei* (Sinton, 1928)

Beni Hassan: April.

*Sergentomyia (Sintonius) tiberiadis* (Adler, Theodor & Lourie, 1930)

Al-Mandaq: March to November.

**Family: Simuliidae**

**Subfamily: Simuliinae**

**Tribe: Simuliini**

*Simulium nili* Gibbins, 1934

Baljurashi: April.

W.Shumrukh: April.

**Family: Tipulidae**

**Subfamily Tipulinae**

**Tribe: Tipulini**

*Tipula* sp.

Ghabet Raghdan: November.

**Suborder: Brachycera**

**Family: Asilidae**

**Subfamily: Laphriinae**

**Tribe: Ctenotini**

*Lamyra vorax* Loew, 1858

Ghabet Shahba: June.

**Family: Bombyliidae**

**Subfamily: Bombyliinae**

**Tribe: Bombyliini**

*Anastoechus trisignatus* (Portschinsky, 1881)

Ghabet Raghdan: May-June.

*Bombylius pallidipilus* Greathead, 1967

Ghabet Raghdan: May-June.

Ghabet Shahba: May-June.

**Subfamily: Toxophorinae**

**Tribe: Gerontini**

*Geron* sp.

Ghabet Shahba: May-june.

Ghabet Raghdan: May-June.

**Subfamily: Anthracinae**

**Tribe: Anthracini**

*Anthrax alruqibi* El-Hawagrysp. n.

Al-Mekhwa: March.

Aqabet Al-Baha-Tihama: April.

Ghabet Raghdan: May.

Ghabet Shahba: June.

*Anthrax chionanthrax* (Bezzi, 1926) [A new record in Saudi Arabia]

Al-Mekhwa: March-April.

*Anthrax ricardoae* Greathead, 2003

Baljurashi: September.

Ghabet Raghdan: June.

Ghabet Shahba: May-June.

*Anthrax sticticus* Klug, 1832

Al-Mekhwa: March-April.

*Spogostylum dagomba* (Bowden, 1964)

Aqabet Al-Baha-Tihama: April-May.

*Spogostylum ocyale* (Wiedemann, 1828)

Al-Mekhwa: April.

*Spogostylum niphas* Hermann, 1907

Al-Mekhwa: April-May.

*Spogostylum* near *tripunctatum* Pallas *in* Wiedemann, 1818 [A new record in Saudi Arabia]

Al-Mekhwa: March-April.

Aqabet Al-Baha-Tihama: April-May.

Ghabet Shahba: June.

**Tribe: Aphoebantini**

*Cononedys dichromatopa* (Bezzi, 1925) [A new record in Saudi Arabia]

Al-Mekhwa: April-May.

Aqabet Al-Baha-Tihama: April.

*Cononedys inornata* (Greathead, 1967)

Al-Mekhwa: April-May.

Aqabet Al-Baha-Tihama: April.

**Tribe: Exoprosopini**

*Exoprosopa disrupta tihamae* Greathead, 1980

Al-Mekhwa: March-April.

*Exoprosopa efflatouni* Bezzi, 1925

Al-Mekhwa: March-May.

*Exoprosopa eritreae* Greathead, 1967

Al-Mekhwa: April-May.

*Exoprosopa pharaonis* Paramonov, 1928

Al-Mekhwa: March-May.

*Exoprosopa pusilla* Macquart, 1840

Al-Mekhwa: March-May.

*Heteralonia (Homolonia) aegina* (Wiedemann, 1828)

Aqabet Al-Baha-Tihama: May.

*Heteralonia (Homolonia) megerlei* (Meigen, 1820)

Beni Hassan: June.

Ghabet Raghdan: May-June.

*Litorhina metapleuralis* Bezzi, 1924

Adnan (near El-Mandaq): September.

*Ligyra astarte* Greathead, 1980

Al-Mekhwa: January-February.

*Ligyra monacha* (Klug, 1832)

Al-Mekhwa: January-February.

Ghabet Raghdan: May-June.

*Ligyra virgo* (Bezzi, 1924)

Ghabet Raghdan: May-June.

*Micomitra chrystallina* Bezzi, 1924

Baljurashi: September.

*Pachyanthrax circe* (Klug, 1832)

Ghabet Raghdan: May-June.

*Pterobates chalybaea* (Röder, 1887)

Ghabet Raghdan: May-June.

**Tribe: Villini**

*Caecanthrax arabica* (Macquart, 1840)

Ghabet Shahba: June.

*Exhyalanthrax afer* (Fabricius, 1794)

Aqabet Al-Baha-Tihama: April.

*Exhyalanthrax beckerianus* (Bezzi, 1924)

Ghabet Raghdan: May.

*Exhyalanthrax triangularis* Bezzi, 1924

Aqabet Al-Baha-Tihama: April.

*Villa cana* (Meigen, 1804)

Ghabet Raghdan: May.

*Villa paniscoides* Bezzi, 1912

Jebel El-Baher: June.

**Tribe: Xeramoebini**

*Petrorossia letho* (Wiedemann, 1828)

Ghabet Shahba: May-June.

*Petrorossia tropicalis* Bezzi, 1921

Ghabet Shahba: May-June.

*Xeramoeba semirufa* (Sack, 1909)

Ghabet Shahba: May-June.

### 

#### Anthrax alruqibi

El-Hawagry sp. n.

urn:lsid:zoobank.org:act:5CF1182F-656F-4EB1-929C-CD2BA4E7CB4C

http://species-id.net/wiki/Anthrax_alruqibi

Figs 2 [Fig F3] 4

##### Remarks.

This species resembles *Anthrax tureus* Greathead, 1980 in size, vestiture, and venation. However, it differs in having faint brownish spots on r-m crossvein, on the origin of vein R_2+3_, on the middle of cell br slightly after origin of vein R_1_, and another fainter and smaller spot may be present on crossvein bm-cu. It differs also in having the wing very feebly tinged brownish at the base. Further, the sides of the 2^nd^ and 4^th^ tergites have tufts of long blackish scales and scaly hairs. The epiphallus terminates in a forceps-like process slightly inclined dorsally and continued with a long flange directed ventrally.

##### Key to the Arabian species of genus *Anthrax* Scopoli.

**Table:** 

1	Wing entirely hyaline, without any infuscated pattern; scales on abdomen mostly white; length 8mm	*Anthrax tureus* Greathead, 1980
–	Wing with an infuscated pattern composed either of a dark blackish brown infuscation on at least the basal third, or with spots on the cross-veins; scales on abdomen mostly black; length 8mm or more	2
2	Wing pattern composed of spots on cross-veins and with only costal cell and bases of basal cells brownish	3
–	Wing pattern composed of extensive basicostal infuscation or numerous irregular blackish brown confluent spots	4
3	Wing with brown spots on cross-veins, origin of R_2+3_ and fork of R_4+5_; sides of abdominal tergites (except the 1^st^) with black hairs; gonocoxites truncate without long posterior processes; length about 10mm	*Sticticus* Klug, 1832
–	Wing with spots on cross-veins and origin of R_2+3_ faint brown, fork of R_4+5_ without a spot; sides of 3^rd^ abdominal tergite with tufts of long snowy whitish scales and scaly hairs, and sides of 3 last tergites with long white hairs seen lower to the black bristles, length about 8mm	*Anthrax alruqibi El-Hawagry* sp. n.
4	Wing pattern very dark blackish-brown with a clear-cut margin	5
–	Wing pattern brown with a diffuse margin merging with darker spots on cross-veins	*Anthrax decisus* Bezzi, 1924
5	Clear area with one or two small isolated spots	*Anthrax aygulus* Fabricius, 1805
–	Clear area without isolated spots	*Anthrax fuscipennis* Ricardo, 1903

##### Etymology.

A patronymic name (A. alruqibi) is proposed in honor of Dr Saeid Al-Ruqib, dean of scientific research in Al-Baha University, Saudi Arabia.

##### Description.

*Holotype male*. Dull black medium sized species. Body length: 8 mm. Wing length: 9 mm.

**Figure 2.**
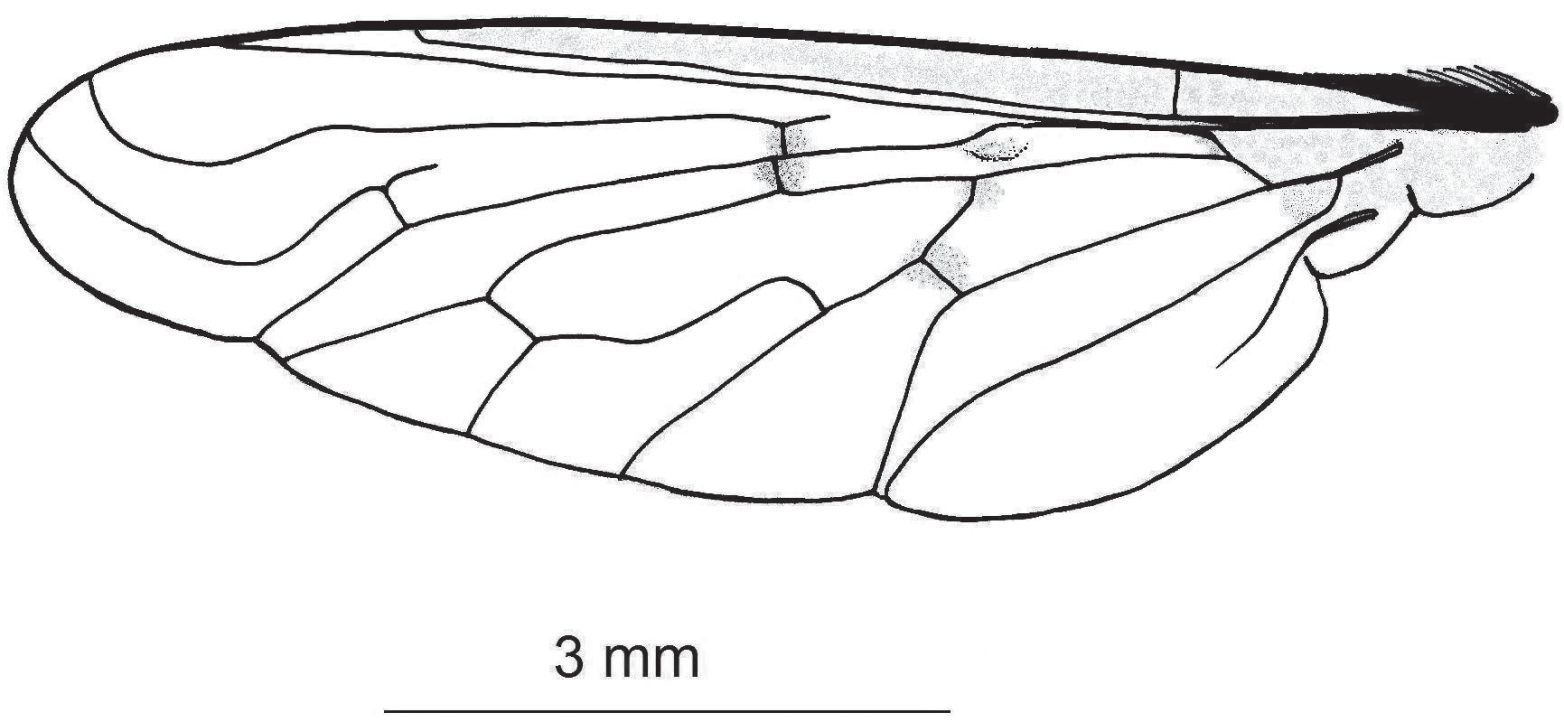
Wing of *Anthrax alruqibi* El-Hawagrysp. n.

**Figure 3. F3:**
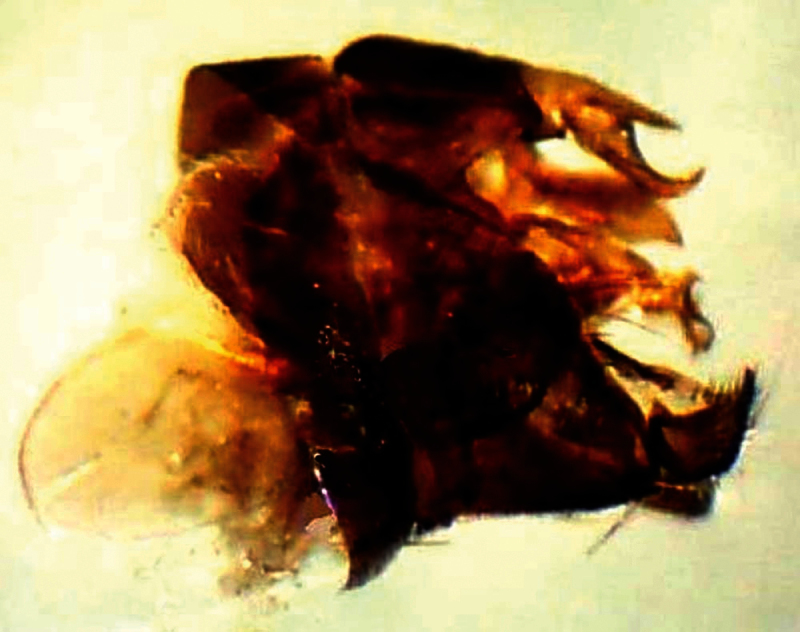
Male genitalia of *Anthrax alruqibi* El-Hawagrysp. n.

**Figure 4.**
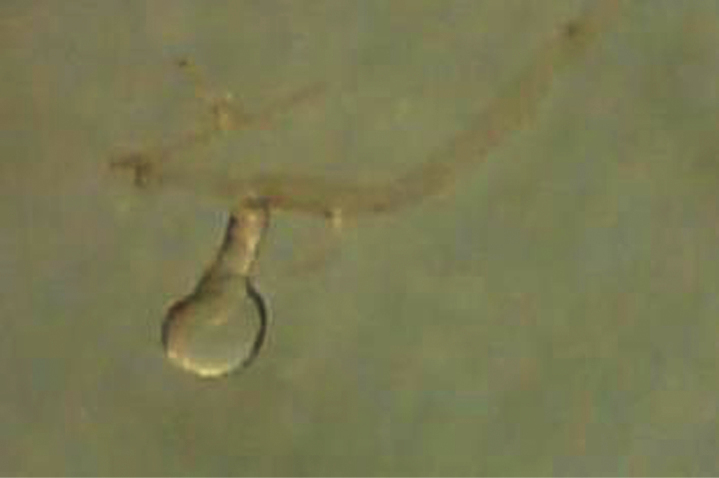
Spermatheca of female *Anthrax alruqibi* El-Hawagrysp. n.

*Head*: Frons with whitish pruinose, tending to be silvery at margins, covered with black hairs, and yellowish to brownish scales at the middle, and the scales become longer, more dense and pale above the antennae; ocellar tubercle black; occiput with whitish pruinose, whitish scales at eye margin, short sparse black hairs becoming more dense behind the ocellar tubercle, and long brownish scaly hairs around the occipital cavity; face covered with whitish long scaly hairs and long black hairs; eyes at upper part of frons separated by about twice width of ocellar triangle; antennae black with some pale brownish pruinose. *Thorax*: Scutum and scutellum covered with fine white and yellowish to brownish white scaly hairs; bristles and hairs black; anterior corners with snowy white scaly hairs, being shaggy and more slender at fore margin; hind margin of scutellum with short white scales; legs black; hairs and bristles black; coxae and tibiae covered with white scales, mixed with brownish white ones on tibiae; claws black; pulvilli grayish; wing hyaline (Fig. 2) with a feeble basicostal infuscation, with a faint brownish spots on r-m, on the origin of vein R_2+3_, on the middle of cell br slightly after origin of vein R_1_, and another fainter and smaller one may present also on bm-cu crossvein; squama with a short white fringe; plumula white; coastal hook black with white scales; halteres brown with knobs white at tip. ***Abdomen***: Corners of 1^st^ tergite with snowy whitish tuft of long scaly hairs; sides of 2^nd^ and 4^th^ tergites with tufts of long blackish scales and scaly hairs; sides of 3^rd^ tergite with tufts of long snowy whitish scales and scaly hairs; bristles of abdomen black and strongly developed; sides of 3 last tergites with long white hairs seen lower to the black bristles; posterior margin of all tergites with snowy whitish scales, becoming more dense and broad at sides especially at sides of 6^th^ tergite; yellowish white scaly hairs and small scales present across mid-line of 2^nd^, 3^rd^, and 4^th^ tergites; tergites with dense black scales lying flat especially on sides of 4^th^ and 5^th^ ones. ***Hypopygium*** (Fig 3): Posterior processes of gonocoxites long and narrow; epiphallus terminating in a forceps-like process slightly inclined dorsally and continued with a long flange directed ventrally. ***Patatype female***. Similar to holotype male; spermatheca (Fig. 4) weakly sclerotized, with globular capsules, ejection apparatus short.

##### Distribution.

**Holotype male**, Aqabet Al-Baha-Tihama, Al-Baha Province, Saudi Arabia (20.00000°N, 41.43758°E, 1300 m.a.s.l.), 18-19.IV.2012 (El-Hawagry ). Paratypes: 1 female, the same holotype data; 1 male, Al-Mekhwa, 21.III.2012 (El-Hawagry); 1 male, Ghabet Raghdan, 12.V.2012 (El-Hawagry); Ghabet Shahba, 8.VI.2012 (El-Hawagry). Holotype and paratypes are deposited in Efflatoun collection, Entomology Department, Faculty of Science, Cairo University, Egypt (**EFC**).

### 

**Family: Mydidae**

**Subfamily: Mydinae**

*Mydas* sp. [A new record in Saudi Arabia]

Al-Mekhwa: April.

**Family: Tabanidae**

**Subfamily: Tabaninae**

**Tribe: Tabanini**

*Tabanus mordax* Austen, 1911

Al-Baha: July.

**Tribe: Haematopotini**

*Haematopota coronata* Austen, 1908

Al-Mandaq: April.

Jebel Ibrahim: September.

Wadi Diyhan: September.

*Haematopota* sp.

Wadi Diyhan: May.

Wadi Gala: May.

**Family: Therevidae**

**Subfamily: Therevinae**

*Thereva* sp.

Al-Mekhwa: January-March.

Dhee Ain: April.

**Suborder: Cyclorrhapha**

**Section: Aschiza**

**Family: Syrphidae**

**Subfamily: Eristalinae**

**Tribe: Eristalini**

*Eristalis taeniops* Wiedemann, 1818

Ghabet Raghdan: May-June.

Al-Baha (Jebel El-Baher): June.

**Tribe: Milesiini**

*Chalcosyrphus* sp.

Ghabet Raghdan: May-June.

Al-Baha (Jebel El-Baher): June.

**Subfamily: Syrphinae**

**Tribe: Syrphini**

*Eupeodes corollae* (Fabricius, 1794)

Ghabet Raghdan: May-June.

Al-Baha (Jebel El-Baher): June.

**Section: Schizophora**

**Subsection: Acalyptratae**

**Family: Chloropidae**

*Oscinella (Cyclocercula) nartshukiana* Beschovski, 1978

Baljurashi: May.

*Oscinella (Paroscinella) acuticornis* Becker, 1912

Baljurashi: May.

*Polyodaspis robusta* (Lamb, 1918)

Al-Mekhwa: March.

*Lagaroceras sequens* Becker, 1910

Al-Baha: June.

**Family: Diopsidae**

*Diopsis apicalis* Dalman, 1817

Al-Mekhwa: December-February.

Dhee Ain: February-May.

*Sphyracephala beccarii* (Rondani, 1873)

Al-Mekhwa: December-February.

Dhee Ain: February-May.

**Family: Drosophilidae**

**Subfamily: Drosophilinae**

**Tribe: Drosophilini**

*Drosophila melanogaster* Meigen, 1830

Common species.

*Drosophila* sp.

Al-Baha (Al-Hawya): May-June.

**Family: Milichiidae**

**Subfamily: Madizinae**

*Desmometopa varipalpis* Malloch, 1927

Al-Mekhwa: March

**Family: Tephritidae**

**Subfamily: Dacinae**

**Tribe: Dacini**

*Dacus frontalis* Becker, 1922

Al-Mekhwa: February.

Dhee Ain: September.

*Dacus vertebratus* Bezzi, 1908

Dhee Ain: September.

*Bactrocera zonata* (Saunders, 1842)

Al-Mekhwa: February.

Baljurashi: September.

Beni Hassan: August.

Dhee Ain: September.

**Subfamily: Tephritinae**

**Tribe: Noeetini**

*Ensina sonchi* (Linnaeus, 1764) Host plant: Asteraceae

Ghabet Raghdan: June.

**Tribe: Tephritini**

*Capitites augur* (Frauenfeld, 1857) Host plant: *Pulicaria Arabica*

Al-Mekhwa: May.

**Family: Ulidiidae**

**Subfamily: Ulidiinae**

**Tribe: Ulidiini**

*Physiphora ?alceae* (Preyssler, 1791)

Al-Mekhwa: February.

**Subsection: Calyptratae**

**Family: Anthomyiidae**

**Subfamily Anthomyiinae**

**Tribe: Anthomyiini**

*Anthomyia benguellae* Malloch, 1924

Ghabet Amadan: May.

Wadi Turabet Zahran: October.

**Family: Calliphoridae**

**Subfamily: Calliphorinae**

*Calliphora vicina* (Robineau-Desvoidy, 1830)

Al-Baha (Jebel Al-Baher): February to July.

**Subfamily: Chrysomyinae**

*Chrysomya albiceps* (Wiedemann, 1819)

Al-Baha City: September.

Wadi Turabet Zahran: May.

*Chrysomya regalis* Robineau-Desvoidy, 1830

Wadi Galla: May.

Wadi Turabet Zahran: May.

**Subfamily: Luciliinae**

*Lucilia sericata* (Meigen, 1826)

Wadi Turabet Zahran: May.

**Subfamily: Polleniinae**

*Pollenia hungarica* Rognes, 1987

Wadi Dahyan: May.

Wadi Turabet Zahran: May.

**Subfamily: Rhiniinae**

*Cosmina viridia* Townsend, 1917

Wadi Galla: May.

Wadi Genouna.

**Family: Hippoboscidae**

**Subfamily Hippoboscinae**

*Hippobosca camelina* Leach, 1817

All localities (on camels): Throughout the year

*Hippobosca equina* Linnaeus, 1758 [? A new record in Saudi Arabia]

Al-Baha [Al-Maslakh] (on cattle): Throughout the year

*Hippobosca longipennis* Fabricius, 1805

All localities (on dogs): Throughout the year

*Hippobosca variegata* Megerle, 1803

All localities (on camels and cattle): Throughout the year

**Subfamily Lipopteninae**

*Melophagus ovinus* (Linnaeus, 1758)

All localities (on sheep and goats): Throughout the year

**Family: Muscidae**

**Subfamily: Atherigoninae**

**Tribe: Atherigonini**

*Atherigona humeralis* Wiedemann, 1830

Dhee Ain: October.

*Atherigona* sp.

Al-Mekhwa: April-July.

**Subfamily: Muscinae**

**Tribe: Muscini**

*Musca albina* Wiedemann, 1830

Al-Mekhwa: March-July.

*Musca domestica domestica* Linnaeus, 1758

Common everywhere and all the time.

*Musca lucidula* (Loew, 1856)

Al-Baha (Shahba): April.

**Subfamily: Phaoniinae**

**Tribe: Phaoniini**

*Helina coniformis* (Stein *in* Becker, 1903)

Baljurashi: August.

*Helina lucida* (Stein, 1913)

Baljurashi: March.

**Subfamily: Coenosiinae**

**Tribe: Limnophorini**

*Lispe nivalis* Wiedemann, 1830

Wadi Turabet Zahran: October.

**Tribe: Coenosiini**

*Coenosia humilis* Meigen, 1826

Al-Baha (Jebel El-Baher): April-August

Al-Mekhwa: March-September.

**Family: Oestridae**

*Oestrus ovis* (Linnaeus, 1958)

Al-Maslakh (on sheep): March to August.

*Przhevalskiana silenus* Brauer, 1858

Al-Maslakh (on goats): March to August.

**Family: Sarcophagidae**

**Subfamily: Sarcophaginae**

*Engelisca adhamae* Lehrer and Abou-Zied, 2008

Al-Baha (Jebel Al-Baher): March to August.

*Liosarcophaga babiyari* (Lehrer, 1995)

Al-Baha (Jebel Al-Baher): March to November.

*Sarcophaga dux* Thompson, 1869

Al-Baha (Jebel Al-Baher): February to September.

**Family: Tachinidae**

*Exorista sp*.

Ghabet Shahba: May-July.

* **Collecting methods of specimens of the order Diptera:** Aerial nets, sweeping nets and malaise traps were the main methods. However, other methods were effective too as bait traps for Calliphoridae and Sarcophagidae; yellow pan traps for Chloropidae, Chironomidae and Syrphidae; sticky traps for Psychodidae; and light traps for Ceratopogonidae and Psychodidae.

**Order: Hymenoptera**

**Suborder: Apocrita**

**Family: Agaonidae**

**Subfamily: Otitesellinae**

*Otitesella rotunda* van Noort, 1997

Jebel Ibrahim: ?

**Family: Apidae**

**Subfamily: Apinae**

**Tribe: Apini**

*Apis florae* Fabricius, 1787

Wadi Galla: May-September.

Wadi Turabet Zahran: May-October.

Dhee Ain: May-August.

*Apis mellifera* Linnaeus, 1758

Common everywhere and all the time

**Tribe: Melectini**

*Melecta sinaitica* (Alfken, 1937)

Dhee Ain: May.

**Subfamily: Xylocopinae**

**Tribe: Xylocopini**

*Xylocopa aestuans* (Linnaeus, 1758)

Wadi Turabet Zahran: March-April.

*Xylocopa* sp.

Ghabet Raghdan: May-June.

**Family: Braconidae**

**Subfamily: Braconinae**

**Tribe: Aphrastobraconini**

*Iphiaulax agnathus* Kohl, 1906

Al-Baher: May.

**Family: Crabronidae**

**Subfamily: Bembicinae**

**Tribe: Bembicini**

*Bembix oculata* Panzer, 1801

Jebel El-Baher: June-July.

*Bembix* sp.

Al-Mekhwa: February-April.

**Subfamily: Crabroninae**

**Tribe: Crabronini**

*Dasyproctus arabs* Kohl, 1894

Jebel El-Baher: May-July.

**Subfamily: Philanthinae**

*Cerceris albicincta* Klug, 1845

Ghabet Shahba: June-August.

*Cerceris alboatra* Walker, 1871

Jebel El-Baher: May-August.

*Cerceris sabulosa* Panzer, 1799

Jebel El-Baher: May-August.

*Philanthus triangulum* Fabricius, 1775

Jebel El-Baher: May-August.

**Family: Eumenidae**

*Eumenus* sp.

Wadi Galla: May.

*Eumenes dimidiatipennis* Saussure, 1852

Wadi Turabet Zahran: May-August.

**Family: Formicidae**

**Subfamily: Aenictinae**

*Aenictus arabicus* Sharaf & Aldawood, 2012-12-23

Aqabet Al-Baha-Tihama: April.

**Subfamily: Dolichoderinae**

*Tapinoma wilsoni* Sharaf & Aldawood, 2012

Dhee Ain: May-September.

*Technomyrmex briani* Sharaf, 2009

Ghabet Shahba: May.

*Technomyrmex setosus* Collingwood, 1985

Baljurashi (Al-Qama’): May.

Ghabet Shahba: May.

Wadi El-Zarayeb: May.

**Subfamily: Dorylinae**

*Dorylus* sp.

Wadi Turabet Zahran: May.

**Subfamily: Formicinae**

*Anoplolepis longitarsus* Collingwood & Agosti, 1996

Baljurashi (Al-Qama’): May.

*Camponotus aegyptiacus* Emery, 1915

Wadi Al-Uqdah: August.

Wadi Aridah: September.

Dhee Ain: May.

*Camponotus iglii* Forel, 1894

Wadi El-Zarayed: May.

Dhee Ain: May.

*Camponotus sericeus* Fabricius, 1798

Wadi Aridah: September.

Wadi Dhiyan: September.

Dhee Ain: May.

*Camponotus flavomarginatus* Mayr, 1862

Al-Baha City: May.

Baljurashi (Al-Qama’): May.

Ghabet Raghdan: May.

*Camponotus xerxes* Forel, 1904

Wadi Aridah: October.

*Camponotus* sp.

Amadan: May.

Al-Baha City: May.

Baljurashi (Al-Qama’): May.

Ghabet Raghdan: May.

Ghabet Shahba: May.

Wadi El-Zarayeb.

*Cataglyphis albicans* (Roger, 1859)

Al-Baha City: May.

Baljurashi (Al-Qama’): May.

Ghabet Raghdan: May.

Ghabet Shahba: May.

*Cataglyphis desertorum* (Forel, 1894)

Wadi Aridah: February.

*Cataglyphis emmae* (Forel, 1909)

Al-Baha: March.

*Cataglyphis holgerseni* Collingwood & Agosti, 1996

Al-Baha City: May.

Baljurashi (Al-Qama’): May.

Ghabet Raghdan: May.

Ghabet Shahba: May.

*Cataglyphis niger* (Andre, 1882)

Wadi Arida: March.

*Cataglyphis semitonsa* Santschi, 1926

Al-Baha: March.

*Savignyi savignyi* (Dufour, 1862)

Amadan: May.

Ghabet Raghdan: May.

*Lepisiota canescens* Emery, 1897

Al-Baha: March.

*Lepisiota obtusa* (Emery, 1901)

Amadan: May.

Baljurashi (Al-Qama’): May.

Ghabet Raghdan: May.

Wadi El-Zarayeb: May.

*Lepisiota opaciventris* (Finzi, 1936)

Baljurashi (Al-Qama’): May.

Wadi El-Zarayeb: May.

*Paratrechina longicornis* (Latreille, 1802)

Dhee Ain: May.

**Subfamily: Myrmicinae**

*Carebara abuhurayri* Sharaf & Aldawood, 2011

Dhee Ain: May.

*Crematogaster affabilis* Forel, 1907

Amadan: May.

Al-Baha City: May.

Dhiyan: September.

Baljurashi (Al-Qama’): May.

Ghabet Raghdan: May.

Wadi El-Zarayeb: May.

*Leptothorax angulatus* Mayr, 1862

Aridah: September.

*Leptothorax* sp.

Ghabet Shahba: May.

*Messor ebininus* Santschi, 1927

Amadan: May.

Baljurashi (Al-Qama’): May.

Ghabet Raghdan: May.

Wadi El-Zarayeb: May.

*Messor* sp.

Amadan: May.

Wadi El-Zarayeb: May.

*Monomorium destructor* (Jerdon, 1851)

Dhee Ain: May.

*Monomorium dryhimi* Aldawood & Sharaf, 2011

Amadan: May.

Baljurashi (Al-Qama’): May.

*Monomorium* ?*exiguum* Forel, 1894

Dhee Ain: May.

*Monomorium mayri* Forel, 1902

Amadan: May.

Ghabet Raghdan: May.

Ghabet Shahba: May.

Wadi El-Zarayeb: May.

*Monomorium salomonis* (Linnaeus, 1758)

Amadan: May.

Baljurashi (Al-Qama’): May.

Ghabet Shahba: May.

*Monomorium sarawatensis* Sharaf & Aldawood sp. n.

Aqabet Al-Baha-Tihama: April.

*Monomorium* sp.

Ghabet Shahba: May.

Dhee Ain: May.

*Nesomyrmex angulatus* Mayr, 1862

Baljurashi (Al-Qama’): May.

Dhee Ain: May.

*Pheidole megacephala* (Fabricius, 1793)

Wadi Al-Uqdah: August.

*Pheidole ?sculpturata* Mayr, 1866

Dhee Ain: May.

*Pheidole* sp.

Amadan: May.

Baljurashi (Al-Qama’): May.

Ghabet Raghdan: May.

Ghabet Shahba: May.

Wadi El-Zarayeb: May.

*Solenopsis elhawagryi* Sharaf & Aldawood, 2012

Baljurashi (Al-Qama’): May.

*Strumigenys* sp.

Dhee Ain: September.

*Tetramorium amalae* Sharaf & Aldawood, 2011

Amadan: May.

*Tetramorium caldarium* Roger, 1857

Baljurashi (Al-Qama’): May.

*Tetramorium sericeiventre* Emery, 1877

Amadan: May.

Baljurashi (Al-Qama’): May.

Ghabet Raghdan: May.

Ghabet Shahba: May.

Wadi El-Zarayeb: May.

*Tetramorium latinode* Collingwood & Agosti, 1996

Amadan: May.

*Tetramorium caldarium* (Roger, 1857)

Al-Baha City: May.

Baljurashi (Al-Qama’): May.

*Tetramorium depressiceps* Menozzi, 1933

Amadan: May.

Al-Baha City: May.

Ghabet Raghdan: May.

Ghabet Shahba: May.

*Tetramorium* sp.1

Al-Baha City: May.

Baljurashi (Al-Qama’): May.

*Tetramorium* sp.2

Dhee Ain: May.

**Subfamily: Ponerinae**

*Anochetus traegaordhi* Mayr, 1904

Dhee Ain: September.

**Subfamily: Pseudomyrmecinae**

*Tetraponera bifoveolata* Mayr, 1895

W. Ibrahim: March.

W. Sanakah: September.

### 

#### Monomorium sarawatensis

Sharaf & Aldawood sp. n.

urn:lsid:zoobank.org:act:9E547C91-E6B5-4E42-9DC2-386D846C4167

http://species-id.net/wiki/Monomorium_sarawatensis

**Figures 5–6.**
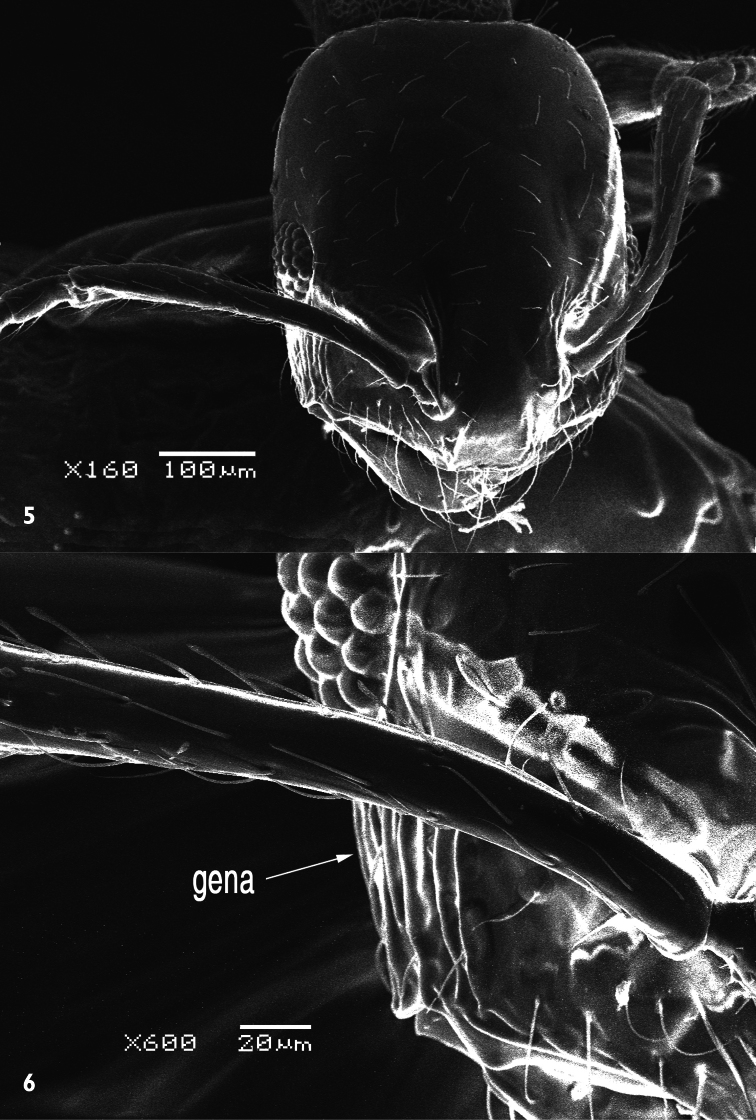
SEM of *Monomorium sarawatensis* sp.n., paratype worker, head in full-face view.

Figs 5 [Fig F6] [Fig F7] [Fig F8] –15

##### Measurements and indices:

TL Total Length; the outstretched length of the ant from the mandibular apex to the metasomal apex.

HW Head Width; the maximum width of the head behind eyes in full face view.

**HL** Head Length; the maximum length of the head, excluding the mandibles.

**CI** Cephalic Index (HW × 100/HL).

**SL** Scape Length, excluding basal neck.

**SI** Scape Index (SL × 100/HW).

**EL** Eye Length; the maximum diameter of the eye.

**ML** Mesosoma Length; the length of the mesosoma in lateral view, from the point at which the pronotum meets the cervical shield to the posterior base of the propodeal lobes or teeth.

**PRW** Pronotal width, maximum width in dorsal view.

**PL** Petiole Length; the maximum length measured in dorsal view, from the anterior margin to the posterior margin.

**PW** Petiole Width; maximum width measured in dorsal view.

**PPL** Postpetiole Length; maximum length measured in dorsal view.

**PPW** Postpetiole Width; maximum width measured in dorsal view.

All measurements are in millimeters and follow the standard measurements (Bolton 1987).

This new species is a member of the *Monomorium monomorium*-group as defined by Bolton (1987), but it does not fit any of the *Monomorium* species in Bolton’s key to the Afrotropical species or the key to the Arabian species given by Collingwood and Agosti (1996). *Monomorium sarawatensis* superficially seems to be similar to *Monomorium affabile* Santschi and *Monomorium malatu* Bolton described from Zaire. The three species share the following characters: dorsum and sides of propodeum and waist blanketed everywhere with dense reticulate-punctate sculpture; fourth (basal) tooth of mandible slightly smaller than the third, and not broadly separated; genae faintly longitudinally striated; body pilosity clubbed. However, *sarawatensis* can be easily separated by the uniform yellow color, whereas the color of the latter species is dark brown to blackish-brown. In comparison with *affabile*, *sarawatensis* is consistently larger (TL 1.77-2.13), versus (TL 1.5) and the eyes are smaller (EL 0.17-0.22 × HW, versus EL 0.24 × HW).

The type locality is a farm planted with *Annona squamosa* L. (Annonaceae), *Prunus persica* (L.), *Prunus Amigdalus* (Mill.) (Rosaceae), *Psidium guajava* L. (Family: Myrtaceae), *Zea mays* ssp. *mays* L. (Family: Poaceae), in addition to banana, and mango. The new species was found nesting inside a woody fruit of *Annona squamosa*. No males or queens were seen.

##### Diagnosis:

This new species is characterized by a combination of the following characters: eyes with five-six ommatidia in the longest row; genae faintly longitudinally striated; metanotal groove deep and broad; propodeal dorsum making a weak obtuse angle with propodeal declivity; mesosoma and waist densely reticulate-punctate; body pilosity clubbed.

##### Key to the Arabian species of the *Monomorium monomorium*-group

**Table:** 

1	Antennae with 11 segments	2
–	Antennae with 12 segments	5
2	Terminal funicular segment broadly swollen	*Monomorium clavicorne* Andre, 1883
–	Terminal funicular segment enlarged, not Swollen	3
3	Mesosoma without hairs	*Monomorium aeyade* Collingwood & Agosti, 1996
–	Mesosoma with hairs	4
4	Mesonotum with at least six pairs of hairs, two on pronotum, four on mesonotum; antennal scapes shorter (SI 74-84); CI higher (74–80)	*Monomorium exiguum* Forel, 1894
–	Mesosoma with fewer hairs, one pair on pronotum and two one mesonotum; antennal scapes slightly longer (SI 90); CI smaller (71)	*Monomorium baushare* Collingwood & Agosti, 1996
5	Mesosoma and waist densely and conspicuously reticulate-punctate	*Monomorium sarawatensis* sp. n.
–	Mesosoma and waist smooth and shining	6
6	Head, in full-face view, with long hairs surrounding posterior margin and head sides forming a fringe; metanotal groove shallow	*Monomorium qarahe* Collingwood & Agosti, 1996
–	Head, in full-face view, without a fringe of long hairs; metanotal groove sharp and distinct	7
7	Larger yellow species; TL 1.70–2.30, HW 0.40; metanotal groove sharp but too small to break the dorsal outline; pronotum with a single pair of curved hairs	*Monomorium montanum* Collingwood & Agosti, 1996
–	Smaller yellowish to light brownish yellow species, first and second gastral tergites with light brownish bands; TL 1.42–1.84; HW 0.32–0.36; metanotal groove sharp and distinctly breaks the dorsal outline; anterior pronotal margin with two pairs of hairs, middle part of pronotum with a single pair	*Monomorium dryhimi* Aldawood & Sharaf, 2011

##### Description.

Measurements: Holotype worker.TL1.98, HL 0.52, HW 0.42, SL 0.38, ML 0.56, EL 0.08, PRW 0.25, PL 0.14, PW 0.12, PPL 0.11, PPW 0.14, SI 90, CI 81.

***Paratypes***.TL1.77-2.13, HL 0.48-0.53, HW 0.36-0.42, SL 0.30-0.39, ML 0.45-0.56, EL 0.07-0.08, PRW 0.21-0.25, PL 0.09-0.14, PW 0.09-0.12, PPL 0.08-0.11, PPW 0.11-0.14, SI 81-95, CI 75-84.

(N=12).

***Holotype worker*.** Head distinctly longer than broad, with a nearly straight posterior margin and shallowly convex sides; head dorsum smooth and shining with few scattered hair-pits; anterior clypeal margin feebly concave between a pair of obtusely projecting angles which separate anterior and lateral margins; clypeal carinae broadly separated and subparallel; eyes with five-six ommatidia in the longest row (EL 0.17-0.22x HW). With head in profile the posterior margins of eyes at the midlength of sides; antennal scapes, when laid back from their insertions, failing to reach posterior margin of head; genae faintly longitudinally striate. Mesosoma in lateral view with the promesonotum straight or feebly convex; metanotal groove deep and broad; propodeal dorsum making a weak obtuse angle with propodeal declivity; mesosomal pilosity few and sparse, two pairs of erect setae on pronotum, five or more on mesonotum, three on propodeum; propodeal spiracle small and pinhole-like; mesosoma densely reticulate-punctate except for pronotal sides which are nearly smooth and shining. Petiolar node high and acuminatein profile, usually with two pairs of erect setae, petiolar peduncle thick and short. Postpetiole in dorsal view clearly broader than long. Petiole and postpetiole densely reticulate-punctate. Color uniformly yellow. Body pilosity clubbed.

##### Distribution.

*Holotype worker*, Aqabet Al-Baha-Tihama, Al-Baha Province, Saudi Arabia (20.00000°N, 41.43758°E, 1300 m.a.s.l.), 19.IV.2012 (M. R. Sharaf ), deposited in King Saud Museum of Arthropods (**KSMA**), College of Food and Agriculture Sciences, King Saud University, Riyadh, Kingdom of Saudi Arabia.

**Paratypes**. 33 workers, same locality and data as holotype; 1 deposited in the Muséum ďHistoire Naturelle, Geneva, Switzerland (Dr Bernhard Merz); 1 in Naturhistorisches Museum, Basel, Switzerland (Mrs. Isabelle Zürcher-Pfander); 1 in California Academy of Science (Dr Brian Fisher); 1 in the Museum of Comparative Zoology, Harvard University, Cambridge, USA (Prof. E. O. Wilson); 1 in the Division of Entomology (Snow Entomological Collections), University of Kansas Natural History Museum, Lawrence, Kansas, USA (Prof. Michael S. Engel); 1 in World Museum Liverpool, Liverpool, U.K (Mr. Tony Hunter), 1 in The Natural History Museum, London (Mr. Barry Bolton); the remaining paratypes are in the King Saud Museum of Arthropods, King Saud University, Riyadh, Saudi Arabia.

##### Note.

Specimens were photographed by Erin Prado using a JVC KY-F70B 3CCD digital camera attached to a Leica M420 stereomicroscope. All digital images were processed using Auto-Montage (Syncroscopy, Division of Synoptics Ltd, USA) software. Images of the specimens are available in full color on www.antweb.org.

**Figures 7–8. F6:**
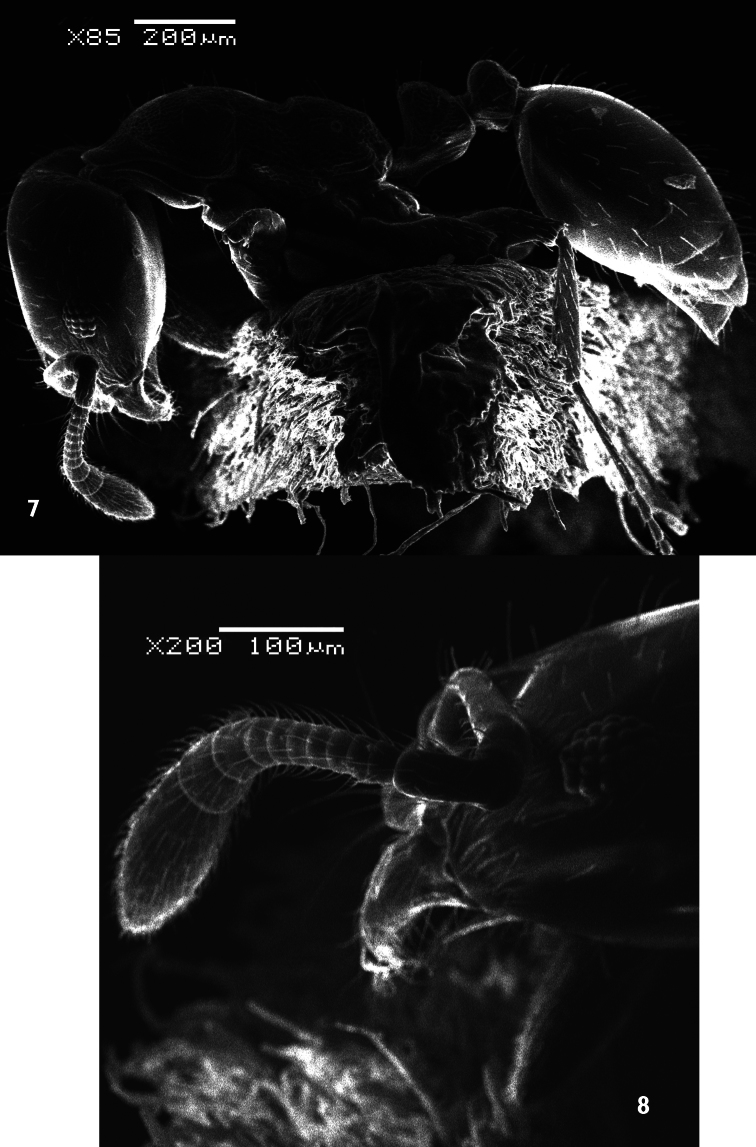
SEM of *Monomorium sarawatensis* sp.n., paratype worker **7** body in profile, 8 head in profile.

**Figures 9–10. F7:**
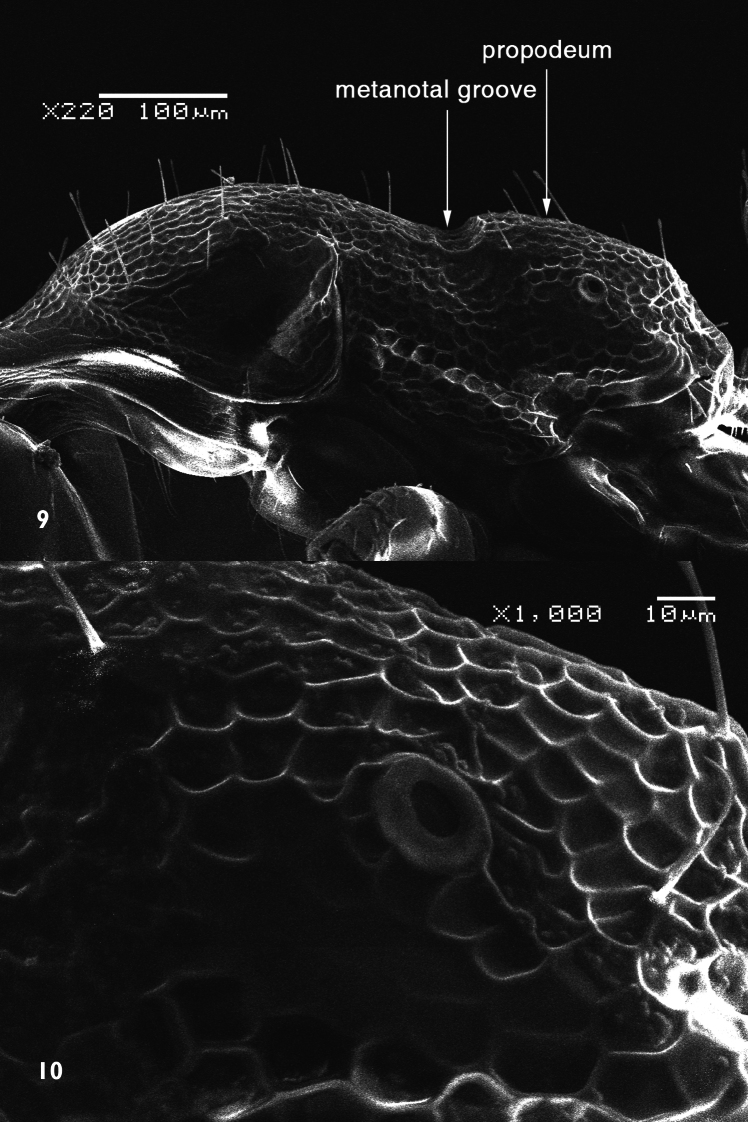
SEM of *Monomorium sarawatensis* sp.n., paratype worker **9** mesosoma in profile **10** propodeal spiracle.

**Figures 11–12. F8:**
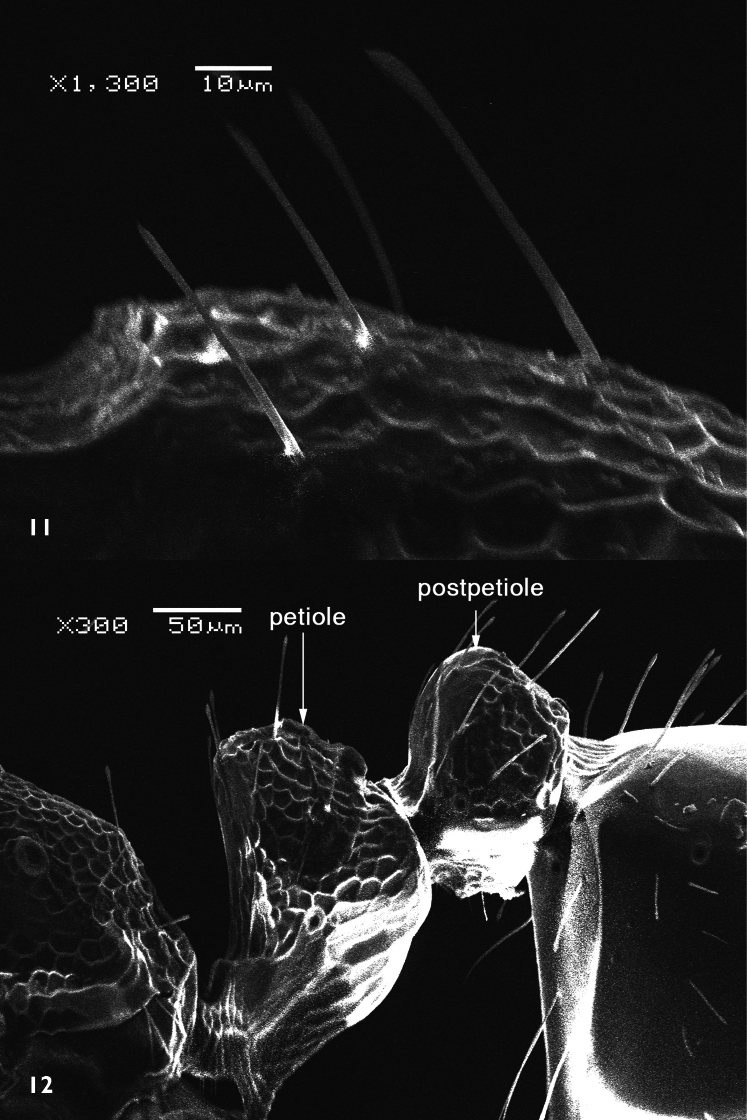
SEM of *Monomorium sarawatensis* sp. n., paratype worker **11** clubbed hairs **12** petiole and postpetiole.

**Figures 13–15.**
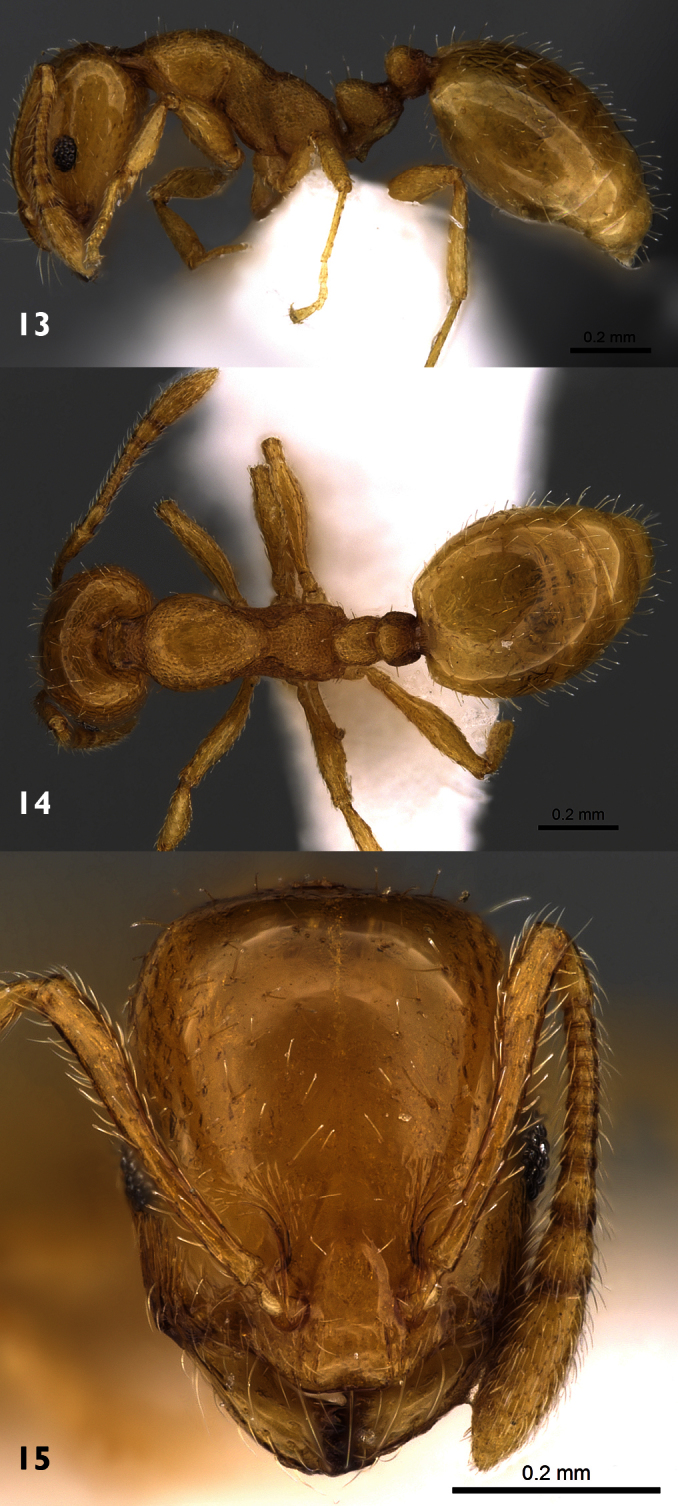
Automontage of *Monomorium sarawatensis* sp.n., paratype worker **13** body in profile **14** body in dorsal view **15** head in full-face view.

### 

Family: Ichneumonidae

**Subfamily: Pimplinae**

*Pimpla* sp.

Jebel El-Baher: May-June.

**Family: Pompilidae**

**Subfamily: Pepsinae**

**Tribe: Pepsini**

*Cyphononyx bretonii* (Guérin, 1843)

Wadi Turabet Zahran: May.

**Family: Scoliidae**

**Subfamily: Campsomerinae**

**Tribe: Campsomerini**

*Micromeriella hyalina* (Klug, 1832)

Gebel El-Baher: May.

*Campsomeriella collaris* (Fabricious, 1775)

Dhee Ain: May-July.

*Campsomeriella thoracica* (Fabricius, 1787)

Al-Baha City (Jebel El-Baher): May.

**Subfamily:** **Scoliinae**

*Scolia* sp.

Al-Mekhwa: March.

Al-Baha City (Jebel El-Baher): May.

**Family: Sphecidae**

**Subfamily: Ammophilinae**

*Ammophila arabica* Kirby, 1900

Al-Mekhwa: March.

Jebel El-Baher: May-August.

*Ammophila erminea* Kohl, 1901

Jebel El-Baher: May-August.

*Podalonia tydei* (Le Guillou, 1841)

El-Baha: June.

**Subfamily: Sphecinae**

*Sphex fumicatus* Christ, 1791

Ghabet Raghdan: June.

**Family: Vespidae**

**Subfamily: Eumininae**

*Delta hottentotum elegans* (De Saussure, 1852)

Ghabet Raghdan: June.

*Delta dimidiatipenne* (de Saussure, 1852)

Ghabet Raghdan: June.

**Subfamily: Polistinae**

**Tribe: Ropalidiini**

*Belonogaster juncea juncea* (Fabricius, 1781)

Gebel El-Baher: May.

**Subfamily: Vespinae**

*Vespa orientalis* Linnaeus, 1771

Wadi Turabet Zahran: May-August.

* **Collecting methods of specimens of the order Hymenoptera:** Aerial nets, sweeping nets and malaise traps were the main methods; however, the yellow pan traps were effective for small Hymenoptera as well, and ants (Formicidae) were collected using tray sifting.

### Faunal richnessand Zoogeographic affinities

25% of the known faunal richness has been accounted for by order Lepidoptera, Diptera comprise 22%, Coleoptera 18%, Hymenoptera 14%, Hemiptera 7%, and Orthoptera 6%. The other insect orders made up 8% of all recorded species.

**Figure 16.**
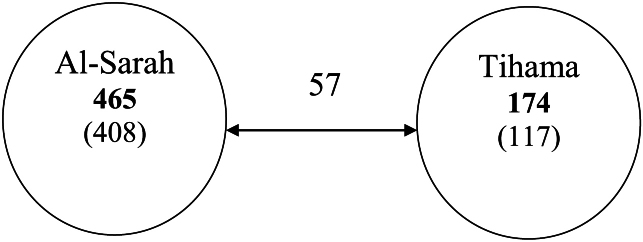
Insect species in the two main sectors (Tihama and Al-Sarah) of Al-Baha Province. The total number of species in each sector is given in bold, the number of species occurring in common in the two sectors is given along the line joining them, and the number of species unique to each sector is given within parentheses within circles.

Insect species richness in Al-Baha Province has been compared between sectors, and with the total species richness in the province as a whole. Results demonstrated that the two sectors (Tihama and Al-Sarah) are varied in their species composition (Fig. 16). The figure summarizes variation in species composition in two ways: firstly, by the number of species shared between the two sectors, and secondly, by the number of species unique to each sector. It was found that 465 species have been recorded from Al-Sarah, with 408 of them (88%) unique; while 174 species have been recorded from Tihama, with 117 of them (67%) unique. However, only 57 species have been recorded as common to both sectors, representing only about 10% of all species recorded from the province as a whole. These results clearly suggest that each of the two sectors of Al-Baha Province (Tihama and Al-Sarah) has its own insect community.

Most of insect species here recorded from Al-Baha Province are characteristic of the Afrotropical region. Tabel (1) indicates the broad scale distribution patterns suggesting a closer affiliation to the Afrotropical region than to the Palearctic region or the Eremic zone. This affiliation was obviously greater in Tihama (69%) than in Al-Sarah (60%). The study showed Palaearcic elements comprising 27% or less in both sectors, in addition to some few Oriental elements (3% or less).

**Table 1.** Zoogeographic affinities of insect species of Al-Baha Province.

**Region**	**Affinities (%)**	
	**Tihama sector**	**Al-Sarah sector**
Afrotropical	69	60
Palaearctic	23	27
Oriental	2	3
Undetermined	6	10

## Discussion

The south-western part of Saudi Arabia, including Al-Baha Province, is considered by many authors to be the most important part of the country and the Arabian Peninsula in general in terms of vegetation and speciation. This area is similar to the high altitude mountains of north-eastern and eastern parts of Africa, both floristically and ecologically (Zohary 1973 and Eig 1938).

Insect diversity (richness) shows a positive correlation with plant diversity (El–Moursy et al. 2001), in other words, the species diversity of consumers should depend to some degree upon the diversity, as well as the productivity, of their food resources (Davidson 1977). Hence, the variation in insect richness in the two sectors of Al-Baha Province seems to reflect their varying vegetation patterns. This variation in insect richness could also be a result of the distance (more than 25km) and altitude (more than 1500 m) between the two sectors, where distance and height could affect the ability of species to disperse between sectors (Fisher 1996). Consequently, each of the two sectors has its own insect community. There is also little doubt that abiotic conditions (relative humidity, soil moisture, temperature, etc.) may affect this pattern of insect distribution in Al-Baha Province.

Considering the insect fauna in Al-Baha Province as a whole, we can obviously conclude that Al-Baha has an extraordinary complex and interesting insect fauna. This may be attributed to its geographical position at the junction of two of the world’s main zoogeographical regions: the Afrotropical and the Palaearctic (Hölzel 1998).

The vegetation of Arabian Peninsula is more or less similar to that of the north-eastern and northern parts of the African Continent. So, some present day biogeographers are of the opinion that the biogeographical divisions within the northeastern and eastern parts of Africa should be extended towards east to cover the regions within the Arabian Peninsula too, namely “Afromontane Archipelago”, covering the high altitude regions of the southern Al-Sarawat Mountains (Zohary 1973; Eig 1938).

Indeed, the present preliminary study is not sufficient to draw more than general conclusions about insect zoogeography in Al-Baha Province. However, the insect faunal composition in this region has an Afrotropical flavor as the Afrotropical elements have been predominantly indicated. Consequently, we tend to agree with those boigeographers who believe that parts of the Arabian Peninsula, including Al-Baha Province, should be included in the Afrotropical region rather than in the Palaearctic region or the Eremic zone, but we cannot indicate the northern border of this region exactly. Especially, Zoogeographical regions often have definable boundaries due to physical barriers, such as mountains, deserts, or water. However, where no such barriers exist, each region gradually merges with the next, pockets of one extending some way into the other due to variable environmental conditions. Such transitional zones may themselves have certain definable characteristics and are often classified as distinct regions. The desert between the Palaearctic and Afrotropical regions is one such zone, and is known as the Afroeremic zone (de Lattin 1967), the Eremic zone (Uvarov 1938; Greathead 1980; Larsen 1984) or the Saharo-Arabian subregion (Takhtajan 1986). However, the northern border of the Afrotropical Region was proposed to be along the Tropic of Cancer (Sclater 1858; Wallace 1876).

We think that the exact indication of the northern border of the Afrotropical region requires more study, not only of the insect fauna but also of the flora and other animal faunas in central deserts, south, south-eastern, and south-western parts of Saudi Arabia.

## Supplementary Material

XML Treatment for Anthrax alruqibi

XML Treatment for Monomorium sarawatensis
